# Sesquiterpene Lactones Potentiate Olaparib-Induced DNA Damage in p53 Wildtype Cancer Cells

**DOI:** 10.3390/ijms23031116

**Published:** 2022-01-20

**Authors:** Hugh C. Osborne, Igor Larrosa, Christine K. Schmidt

**Affiliations:** 1 Manchester Cancer Research Centre (MCRC), Division of Cancer Sciences, Faculty of Biology, School of Medical Sciences, Medicine and Health, University of Manchester, 555 Wilmslow Road, Manchester M20 4GJ, UK; hugh.osborne@postgrad.manchester.ac.uk; 2 Department of Chemistry, University of Manchester, Chemistry Building, Oxford Road, Manchester M13 9PL, UK

**Keywords:** sesquiterpene lactones, 2-bromobenzyloxy derivative of dehydrosantonin (BdS), alantolactone (ATL), PARP inhibitor (PARPi), olaparib, reactive oxygen species (ROS), DNA replication stress, cancer

## Abstract

Despite notable advances in utilising PARP inhibitor monotherapy, many cancers are not PARP inhibitor-sensitive or develop treatment resistance. In this work, we show that the two structurally-related sesquiterpene lactones, a 2-bromobenzyloxy derivative of dehydrosantonin (BdS) and alantolactone (ATL) sensitise p53 wildtype, homologous recombination-proficient cancer cells to low-dose treatment with the PARP inhibitor, olaparib. Exposure to combination treatments of olaparib with BdS or ATL induces cell-cycle changes, chromosomal instability, as well as considerable increases in nuclear area. Mechanistically, we uncover that mitotic errors likely depend on oxidative stress elicited by the electrophilic lactone warheads and olaparib-mediated PARP-trapping, culminating in replication stress. Combination treatments exhibit moderately synergistic effects on cell survival, probably attenuated by a p53-mediated, protective cell-cycle arrest in the G2 cell-cycle phase. Indeed, using a WEE1 inhibitor, AZD1775, to inhibit the G2/M cell-cycle checkpoint further decreased cell survival. Around half of all cancers diagnosed retain p53 functionality, and this proportion could be expected to increase with improved diagnostic approaches in the clinic. Utilising sublethal oxidative stress to sensitise p53 wildtype, homologous recombination-proficient cancer cells to low-dose PARP-trapping could therefore serve as the basis for future research into the treatment of cancers currently refractory to PARP inhibition.

## 1. Introduction

DNA damage, resulting from a broad range of endogenous and environmental factors, is tumourigenic if left unchecked. Due to DNA lesion diversity, a series of distinct repair processes has evolved to function in concert towards the repair of this damage [1]. As a whole, these repair processes together with downstream signalling events are termed the DNA damage response (DDR). A proper working of the DDR is critical in the maintenance of genome stability and certain cancers employ an incomplete DDR [2]. Exploiting this difference between healthy and malignant cells by inhibiting a parallel or intersecting repair pathway to selectively induce cancer cell senescence or apoptosis is termed ‘synthetic lethality’. The first clinical utilisation of synthetic lethality was the use of poly(ADP-ribose) polymerase (PARP) inhibition in BRCA (BReast CAncer, early onset)-deficient patients [3]. The use of PARP inhibitors has expanded since their approval in 2014, more recently as a first-line therapy [4]. However, PARP inhibition is currently mostly limited to patient populations displaying certain gene markers and characteristics, and subsequent resistance mechanisms are common. As such, identifying additional strategies to extend the use of PARP inhibitors is of paramount importance [3,4].

PARP1/2 enzymes are key coordinators in the repair of oxidative base damage and single-strand breaks (SSBs) in DNA, associating with DNA prior to decorating themselves with poly(ADP-ribose) (PAR) chains that serve as platforms for further DDR factor recruitment [5]. Canonically, BRCA mutant cancers are considered acutely sensitive to PARP inhibition as unrepaired lesions destabilise replication forks and/or develop into DNA double-strand breaks (DSBs) over the course of the cell cycle [3,5]. A principal DSB repair pathway, homologous recombination (HR), sees break ends undergo nuclease-dependent resection before a sister chromatid is used as a template to complete error-free repair with the help of the RAD51 recombinase, limiting its use to S and G2 phases. BRCA1 and BRCA2 constitute key HR mediators and their dysfunction typically abrogates the repair pathway [6].

In comparing PARP1/2 protein depletion and pharmacological inhibition, not all consequent phenotypes can be rationalised if PARP inhibitor activity were to derive solely from impaired enzyme activity. PARP1 was found to be entrapped by some PARP inhibitors once it binds DNA, at SSBs and unligated Okazaki fragments [4,5,7,8]. Indeed, PARP–DNA complexes, like other steric obstacles to the DNA replication machinery, engender significant replication stress through the accumulation of intermediate replicative structures, DSBs, and incorrectly repaired DSBs [4,5]. Replication stress can leave active and stalled replication forks vulnerable to nucleolytic degradation, following which widespread replication fork collapse occurs (replication catastrophe) [9].

**Figure 1:**
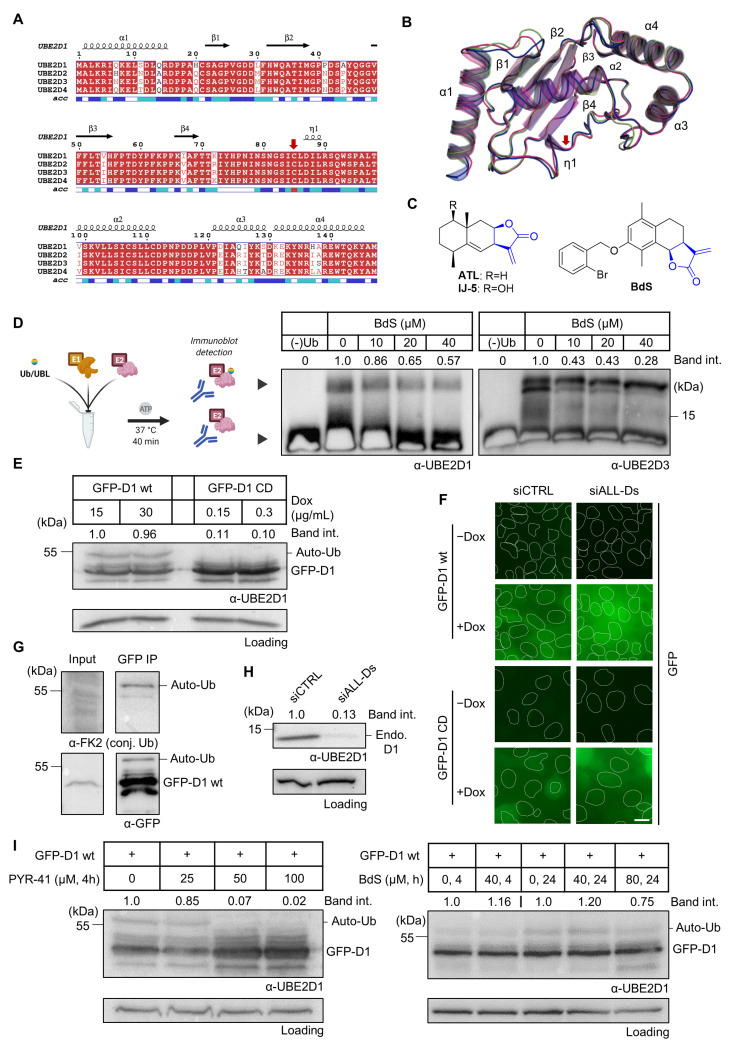
Effects of the sesquiterpene lactone, BdS, on UBE2D activity in vitro and in cells. (**A**) COBALT Alignment and ESPript visualisation of UniProt sequences of UBE2D1-4 [15,16]. Red shading of single-letter amino acid codes indicates homology; white shading shows points of differentiation between enzymes. Red arrow points out catalytic cysteine, C85. Secondary structure elements are displayed at the top for UBE2D1. UBE2D1 accessibility is indicated at the bottom (colour gradient—darker blue to white showing decreasing accessibility). (**B**) Overlay of available UBE2D crystal structures (UBE2D1: PDB 5TUT (red), UBE2D2: PDB 2ESK (green), UBE2D3: PDB 3L1Z (blue)) [17,18,19]. Red arrow indicates catalytic cysteine. (**C**) Chemical structures of sesquiterpene lactones, ATL and IJ-5 (alantolactone, and 1β-hydroxyalantolactone, respectively), and BdS showing their shared covalent-binding warhead in blue. (**D**) Left: In vitro ubiquitylation assay workflow (created using Biorender). Right: Representative blots showing the effects of BdS on ubiquitin-loading for UBE2D1 (*n* = 2 independent experiments) and UBE2D3 (*n* = 3 independent experiments). (**E**) Doxycycline (Dox) induction of GFP-UBE2D1 wt (wild-type) and GFP-UBE2D1 CD (catalytically dead—C85S; *n* = 1 experiment) in U2OS cells. (**F**) Fluorescence images of GFP-UBE2D1 wt and GFP-UBE2D1 CD in siCTRL or siALL-Ds depleted U2OS cells, after doxycycline (Dox) induction or not. Nuclei are outlined in white using DAPI as a reference (scale bar – 20 μm). (**G**) GFP-Trap pulldowns of GFP-UBE2D1 stably expressed in U2OS cells. IP represents 1% of the input (*n* = 2 independent experiments). Immunoblot sections are derived from same membrane. (**H**) siALL-Ds depletion efficiency of endogenous (endo.) UBE2D1 (D1) in U2OS cells stably expressing GFP. Blot is representative of *n* = 4 independent experiments. (**I**) Effects of PYR-41 (left), a ubiquitin E1 (UBA1) inhibitor, and BdS (right) on GFP-UBE2D1 wt auto-ubiquitylation at the indicated concentrations and treatment times (*n* = 1 for PYR-41; *n* = 2 independent experiments for BdS). Normalised blot band intensity quantification was performed using ImageJ.

Possible mechanisms for extending the use of PARP inhibitors could therefore include targeting HR factors upstream of BRCA1/2, such as CtIP, a protein important for initiating DNA end resection at an early stage of HR [10,11]. CtIP accrual to DSBs is promoted by the ubiquitylation of its N-terminus mediated by the UBE2D (UbcH5) family of ubiquitin-conjugating E2 enzymes (Figure 1A,B) [12]. This raises the hypothesis that UBE2D inhibition can hypersensitise cells to PARP inhibitors. Another attractive approach is to amplify S phase PARP-trapping damage by raising the frequency of PARP–DNA complex association, such as via the use of reactive oxygen species (ROS)-inducing small molecules or radiation [13,14]. By eliciting oxidative DNA base damage, ROS generation activates base excision repair (BER), for which PARP1/2 enzymes are early responders, making them amenable for trapping to DNA by suitable inhibitors, such as olaparib and talazoparib [5,14]. The PARP–DNA complexes then lead to an increase in the overall number of replication fork collisions, explaining the hypersensitisation of PARP-trapping inhibitors with ROS-generating small molecules [14].

Sesquiterpene lactones are attractive for both of the above scenarios. They represent a group of small molecules characterised by an α-methylene-γ-butyrolactone warhead. Some sesquiterpene lactones, such as the natural product IJ-5 and its synthetic successor, a 2-bromobenzyloxy derivative of dehydrosantonin (BdS), have been described as UBE2D inhibitors, making them attractive for abrogating HR at an early step by inhibiting CtIP recruitment to DSBs [20,21]. Other analogous compounds, such as alantolactone (ATL), have been implicated in inhibiting the growth of a number of cancer cell lines through ROS induction [14,22,23,24]. The mechanisms through which ATL acts are thought to involve thioredoxin reductase (TrxR) inhibition, a central family of antioxidant enzymes, resulting in synergistic activity with PARP inhibitors [14]. Given the high level of structural similarity between the sesquiterpene lactones indicated in both scenarios, it is critical that we better understand how these compounds exert their functions. Do they mainly act via one pathway or the other, or indeed via both mechanisms-of-action? Moreover, previous work combining PARP-trapping activity with sesquiterpene lactones has exclusively focused on cancer cells featuring impaired p53 function. In this regard, it is interesting to note that olaparib can cause p53-dependent cell-cycle phenotypes, such as the extension of G2 phase [25]. Given the tight link between DNA damage-induced cytotoxicity and cell-cycle progression, the question arises if olaparib combinations with sesquiterpene lactones can be extended to p53 wildtype cancer settings.

Here, we show that the sesquiterpene lactone BdS is efficient in hypersensitising p53 wildtype, HR-proficient cancer cells to low-dose treatments with olaparib. Strikingly, the observed effects are independent of BdS’s inhibitory activity of UBE2Ds, but rather are mediated by BdS potentiating the DNA damage caused by olaparib, similarly to what can be observed with another sesquiterpene lactone, ATL [14]. Mechanistically, the induced DNA damage is dependent on PARP trapping, while pre-treatment with the antioxidant *N*-acetyl cysteine (*N*-AC), and experiments using an inert BdS analogue (BdS-H_2_) establish that BdS activity is conferred by its electrophilic, covalent-binding warhead, and the consequent induction of oxidative DNA damage. Notably, ATL/BdS combination treatments with olaparib are associated with synergistic increases in replication protein A 1 (RPA1) foci, demonstrating increased consumption of the RPA pool that usually protects single-stranded DNA from the action of nucleases, as well as a range of mitotic defects and cell-cycle alterations associated with a global increase in nuclear area. Accordingly, subsequent G2/M checkpoint abolishment using a WEE1 inhibitor further potentiates growth inhibition caused by combination treatments with ATL/BdS and olaparib.

This study provides insights into how early stage, p53 wildtype cancers might be targeted by exploiting oxidative stress to extend the application of PARP inhibition [26]. Independent of RAD51-mediated repair capacity, this work could also prove useful towards the resensitisation of tumours with acquired PARP inhibitor resistance, particularly where replication fork stability is restored [4].

## 2. Results

### 2.1. BdS Activity Is Not Mediated by Cellular Inhibition of UBE2D Enzymes

The UBE2D family is comprised of four ubiquitin E2 enzymes with significant sequence overlap (Figure 1A; sequence identity 87.1%), resulting in highly homologous secondary structures. Four α-helices (α1-4), four β-sheets (β1-4), and a catalytic cysteine (C85) with an adjacent 3_10_-helix (η1, red arrow designating catalytic residue) constitute a core ubiquitin-conjugating (UBC) domain that is found in most E2s (Figure 1A,B) [27]. The homology of the shallow E2 active site across the enzyme superfamily has raised doubts over whether it can be selectively targeted pharmacologically. Prior orthosteric (active site-targeting) E2 pre-clinical inhibitors have mainly consisted of covalent binders, indicative of the UBC domain topology [28]. In 2014, the sesquiterpene lactone IJ-5, a natural product, was identified as a UBE2D1-3 inhibitor, which was further complemented by a later computational study (Figure 1C) [20,29]. Due to the challenging total synthesis of IJ-5, in 2017, a series of α-santonin derivatives were developed and screened for UBE2D activity giving rise to BdS (compound 6d in the referenced work) as a reported UBE2D inhibitor (Figure 1C) [21]. In the presence of BdS, we observed a decrease in in vitro ubiquitin loading of UBE2D3 and to a lesser extent UBE2D1 in a dose-dependent manner, with moderately higher drug concentrations than previously reported (Figure 1D; for synthesis of BdS see Supplementary Figure S1A; for assay control experiments see Supplementary Figure S1C,D). The differential effect of BdS on UBE2D1 and UBE2D3 ubiquitin-loading is concordant with reported data [21].

Based on these findings, we set out to investigate if the observed effects would translate into intracellular UBE2D inhibition. To this end, we utilised U2OS (human osteosarcoma) cells, a model system widely used to study DDR mechanisms, specifically monoclonal U2OS cells, stably expressing doxycycline-inducible wildtype (wt) or catalytically dead GFP-UBE2D1 (active cysteine C85 mutated to serine; C85S) [12]. Doxycycline concentrations were titrated to achieve comparable expression levels of wt and CD GFP-UBE2D1 (Figure 1E). Moreover, wt and catalytically dead GFP-UBE2D1 showed similar distributions across the cytoplasm and nucleus (Figure 1F). As expected, cellular auto-ubiquitylation of GFP-UBE2D1 depended on the catalytic activity of UBE2D1 (Figure 1E,G). Thus, we utilised the cellular auto-ubiquitylation of GFP-UBE2D1 as a system to examine the effects of BdS on intracellular UBE2D1 activity. To ensure that no compensatory UBE2D activity was present in the cells, we depleted endogenous UBE2D enzymes using a previously established siRNA mix (siALL-Ds; Figure 1H). Strikingly, BdS exerted no or only minor effects on GFP-UBE2D1 auto-ubiquitylation at all concentrations and durations tested, while a positive control (PYR-41; ubiquitin E1, UBA1 inhibitor) effectively inhibited it in a concentration-dependent manner (Figure 1I) [30].

**Figure 2:**
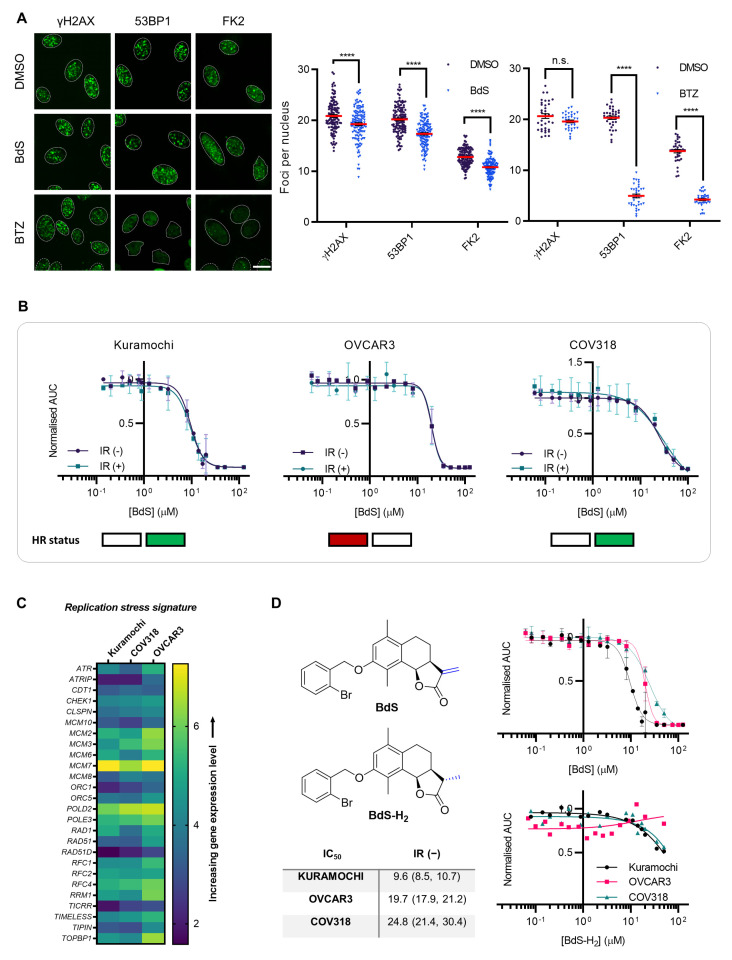
Effects of BdS on DNA repair and cell growth. (**A**) Left: Representative images of U2OS cells treated with BdS, BTZ, or vehicle only (DMSO). Nuclei are outlined in white using DAPI as a reference (scale bar—20 μm). Right: Quantification of foci number per cell for DNA damage response (DDR) factors (γH2AX, 53BP1, FK2; Alexa Fluor 488). For BdS: each dataset represents a minimum of 3000 U2OS cells (3 replicates; *n* *=* 126). For bortezomib (BTZ): each dataset represents a minimum of 3000 U2OS cells (2 independent experiments; *n* = 36). Data points correspond to each recorded field (mean ± SEM). (**B**) Non-linear regression curve analyses of normalised growth curves of Kuramochi (*n* = 3 independent experiments), OVCAR3 (*n* = 3 independent experiments), and COV318 (*n* = 2 independent experiments) cells in the presence of BdS or vehicle only (DMSO) ± irradiation (2 Gγ) across 136 h. Homologous recombination (HR) status of the cells is indicated below (red—deficient, green—proficient) [32,33]. (**C**) Heat map demonstrating differences in gene expression across the indicated cell lines from published data (CCLE) for key replication stress-related genes [34]. (**D**) Top left: comparison of chemical structures of BdS and its inactive analogue, BdS-H_2_ (differing moiety in blue). Bottom left: IC_50_ values calculated from non-linear regression curves shown in (**B**). Values are provided in μM (95% confidence intervals). Top right: Overlay of regression curves shown in (**B**) for non-irradiated samples across the three high-grade serous ovarian carcinoma (HGSOC) cell lines, following treatment with BdS. Bottom right: Overlay of regression curves for non-irradiated samples following treatment with BdS-H_2_. Statistical significance indicated as follows: n.s.—*p* > 0.05, and ****—*p* < 0.0001.

To ensure BdS was not acting via UBE2D inhibition, we next explored if BdS affected UBE2D-dependent cellular phenotypes related to the DDR. To this end, we used quantitative high-content fluorescence microscopy to investigate the recruitment of select DDR proteins to sites of DNA damage induced by ionising radiation (IR, 2 Gγ; Figure 2A and Supplementary Figure S2A). The DDR factors evaluated were γH2AX, a phosphorylated histone variant acting as an upstream sensor of DNA damage; 53BP1, a key DDR factor, whose recruitment to DNA damage sites is ubiquitin- but not UBE2D-dependent; and conjugated ubiquitin (detected by FK2 antibody), which shows decreased foci formation when UBE2Ds are depleted [12]. The proteasome inhibitor bortezomib (BTZ, 0.5 μM) was used as a positive control, resulting in a dearth of ubiquitin available for substrate conjugation [31], and thus, leading to an almost complete abrogation of 53BP1 and FK2 foci formation (Figure 2A). Importantly, γH2AX foci formation remained unchanged, confirming the induction of analogous amounts of DNA damage in DMSO- and BTZ-treated cells, and illustrating the robustness of our experimental pipeline (for additional controls see Supplementary Figure S2B,C). In contrast to BTZ, BdS treatment caused small, phenotypically non-significant reductions in foci formation of conjugated ubiquitin, which did not approach the marked effects achieved previously with siRNA depletion of UBE2Ds (Figure 2A) [12].

### 2.2. Homologous Recombination Status Does Not Dictate BdS Growth Inhibition

High-grade serous ovarian carcinoma (HGSOC) exhibits a high degree of genomic instability, with an elevated frequency of DDR defects, representing a pertinent setting to further scrutinise whether BdS can exacerbate dysfunctional DNA repair [35]. Combining BdS treatment and IR could then demonstrate whether IR hypersensitisation occurred in an HR-competent setting due to BdS-mediated HR inhibition, as would be expected if BdS suppressed CtIP recruitment via the inhibition of UBE2D enzymes. Conversely, HR-deficiency would likely preclude IR sensitisation. To this end, two HR-proficient HGSOC cell lines (Kuramochi, COV318) [32,34] and one HR-deficient cell line (OVCAR3) [33] were subjected to treatments with BdS at varying concentrations (0-125 μM) and in the presence or absence of IR (2 Gγ) (Figure 2B; representative growth curves in Supplementary Figure S2D). All three cell lines expressed GFP-tagged histone H2B (GFP-H2B), which allowed their growth to be accurately tracked by *in situ* fluorescence microscopy using a green object count (GOC) combined with an IncuCyte platform (GOC mask shown in Supplementary Figure S2E) [36]. The non-linear regression curves generated from the cell growth data monitored over approximately 5.5 days (136 h) showed that BdS sensitivity in the IR(+) conditions across all cell lines tightly overlaid with the respective IR(−) curves (Figure 2B). These findings demonstrate that no IR sensitisation was caused by BdS, and further indicate that the compound is unlikely to affect HR by targeting UBE2D enzymes in a range of cancer cell lines tested.

### 2.3. Sensitivity of Ovarian Cancer Cell Lines to BdS Correlates with DNA Replication Stress Profiles

Interestingly, while treatment with BdS did not result in radiosensitisation, it inhibited proliferation to varying extents between the different ovarian cancer cell lines. Unexpectedly, Kuramochi cells appeared to be more than twice as sensitive to BdS than OVCAR3 or COV318 cells, which is reminiscent of the poly(ADP-ribose) glycohydrolase (PARG) inhibitor sensitivity displayed by these cells that correlates with their gene expression-based replication stress signature (Figure 2C) [34,36]. Indeed, the IC_50_ values for BdS in these cell lines (Kuramochi; 9.6 μM, and, OVCAR3; 19.7 μM, COV318; 24.8 μM; Figure 2D) trended coherently with the two HGSOC clusters designated by the signature, in which Kuramochi cells were classified as sensitive, and OVCAR3 and COV318 as resistant [34]. Importantly, a BdS analogue (BdS-H_2_), featuring an inert methyl group in place of the Michael acceptor warhead’s reactive methylene substituent, was unable to significantly inhibit cell growth (Figure 2D; for synthesis and analysis of BdS-H_2_ see Supplementary Figure S1B and Materials and Methods), confirming that BdS-mediated growth inhibition is due to the reactivity conferred by BdS’s electrophilic, covalent-binding warhead. This is in agreement with previous robust findings showing that the absence of the methylene moiety in other sesquiterpene lactones results in the loss of cytotoxicity [37]. With these results in hand, it appears unlikely that BdS inhibits UBE2D-mediated ubiquitylation in the range of model cancer cell lines tested. We also conclude that BdS is unable to extend olaparib usage via the inhibition of UBE2D-dependent DDR processes upstream of BRCA1/2.

### 2.4. BdS Synergises with Olaparib to Induce DNA Damage in p53 Wildtype Cancer Cells

We next set out to test if BdS could extend olaparib usage by other mechanisms. For example, ATL, a sesquiterpene lactone reminiscent in structure to IJ-5 and BdS (Figure 1C), can increase ROS levels in a number of transformed cell lines, which could explain the increased BdS sensitivity of Kuramochi cells given their pre-existing replication stress vulnerability (Figure 2C) [14,38,39,40,41]. Importantly, the increased ROS levels induced by ATL can lead to oxidative DNA damage, marked particularly by the formation of the DNA base oxidation product 8-oxo-7,8-dihydroguanine (8-OxoG), which triggers the induction of base excision repair (BER) [14]. BER is dependent on PARP1/2 enzymes generating PAR chains, which act as docking platforms for downstream DDR proteins.

Combining the base oxidation induced by ATL with olaparib and its known PARP-trapping effects resulted in significant S phase DNA damage due to replication fork collisions with PARP–DNA complexes, leading to apoptosis secondary to the activation of key replication stress proteins and extensive S/G2 arrest [14]. A key limitation arising from these data was the uncertainty of whether the enhanced effects of the combination treatment would apply to p53 wildtype cells, as the main cell lines featured in this context exhibited majorly impaired *TP53* functionality relevant to the studied phenotype [42,43,44,45]. To see if the synergistic growth inhibition observed in the above work would extend to *TP53* wildtype cells, and investigate if BdS could function in an ATL-like fashion to further extend and optimise the usage of olaparib, we evaluated combinations of the drugs with olaparib across a variety of cellular assays in p53 wildtype U2OS cells [14,46,47].

**Figure 3:**
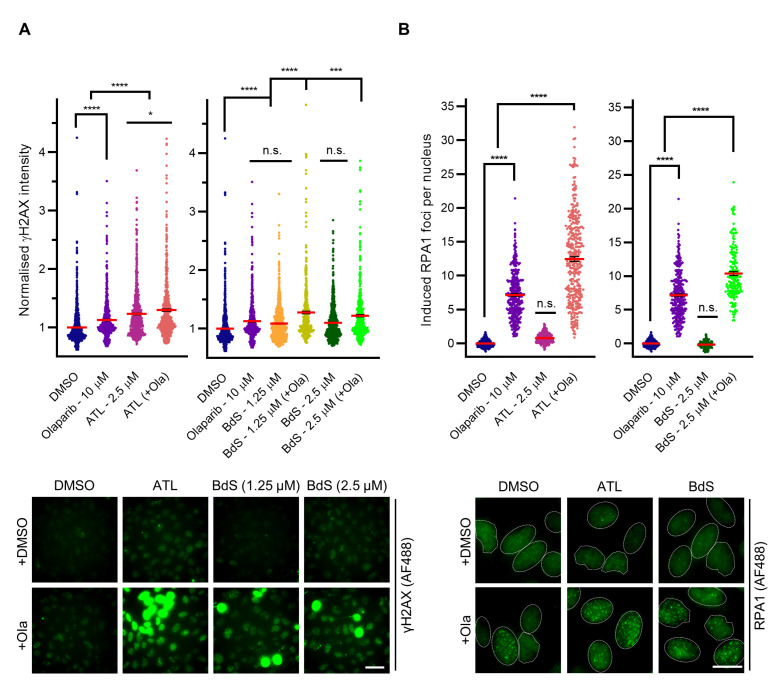
BdS and alantolactone (ATL) potentiate olaparib-mediated DNA damage and replication stress-related RPA consumption. (**A**) Quantification (top) and representative images (bottom) of normalised nuclear γH2AX immunofluorescence intensity (Alexa Fluor 488; AF488) of ≥20,000 U2OS cells (≥3 independent experiments; *n* ≥ 504) following 24 h treatment with olaparib, BdS and/or ATL as indicated, compared to vehicle only (DMSO). Data points correspond to each recorded field (mean ± SEM). DMSO and olaparib control datasets are identical due to forming part of the same experimental pipeline. Scale bar—50 μm. (**B**) Quantification (top) and representative images (bottom) of induced RPA1 foci per cell (Alexa Fluor 488; AF488) of ≥9000 U2OS cells (≥3 replicates; *n* ≥ 168) after 24 h treatment with olaparib, BdS, and/or ATL as indicated, compared to vehicle only (DMSO). Data points relate to each recorded field (mean ± SEM). DMSO and olaparib control datasets are identical due to forming part of the same experimental pipeline. Nuclei are outlined in white using DAPI as a reference. Scale bar—20 μm. Statistical significance indicated as follows: n.s.—*p* > 0.05, *—*p* < 0.05, ***—*p* < 0.001, and ****—*p* < 0.0001.

ATL and BdS were initially titrated to doses that caused minimal increases in DNA damage alone, using nuclear γH2AX intensity as a metric to optimally capture the characteristic pan-nuclear γH2AX staining that occurs following replication catastrophe [36]. When combined with olaparib at a final concentration of 10 μM for 24 h, both ATL and BdS significantly potentiated increases in DNA damage above olaparib alone. Indeed, a lower dose of BdS exacerbated olaparib-induced damage synergistically (1.25 μM; Figure 3A), with further synergistic increases in pan-nuclear γH2AX staining documented across all combination treatments at later time points (72 h; Supplementary Figure S3A,B).

A key rate limiter in the maintenance of replication stress is the available pool of RPA. If RPA is depleted, replication forks can undergo a nuclease-driven collapse at multiple sites across the transcriptome and cause replication catastrophe, which could explain the γH2AX staining observed [9,36]. Therefore, to elucidate a possible mechanism for the induction of DNA damage, we studied RPA1 foci formation after 24 h of drug treatment (Figure 3B). Olaparib treatment alone expectedly induced a significant increase in RPA1 foci, representative of stochastic replication fork collisions with stabilised PARP-DNA complexes during S phase and the resulting resolution of ensuing damage. By contrast, ATL and BdS treatment alone engendered no significant increases in RPA1 foci (Figure 3B). Critically, a strongly synergistic rise in induced RPA1 foci was detected when either of the two compounds were combined with olaparib (Figure 3B), suggesting that the initial rate of RPA consumption is higher in the combination treatments. Importantly, across all sesquiterpene lactone treatments, no change in RPA1 protein level was detected (Supplementary Figure S3C). Increased rates of RPA consumption were followed by synergistic increases in the number of induced 53BP1 foci per cell in these same conditions at 72 h, indicative of significant DSB formation and consistent with replication catastrophe (Supplementary Figure S3D) [9]. Moreover, the resultant γH2AX (Supplementary Figure S4A) and RPA (Supplementary Figure S4B) phenotypes are strongly concordant with those elicited by the combination of a ROS induction positive control compound, tert-butyl hydroperoxide (t-BHP), and olaparib [48].

### 2.5. BdS Exacerbates Olaparib-Mediated PARP-Trapping in a Michael Acceptor-Dependent Manner

**Figure 4:**
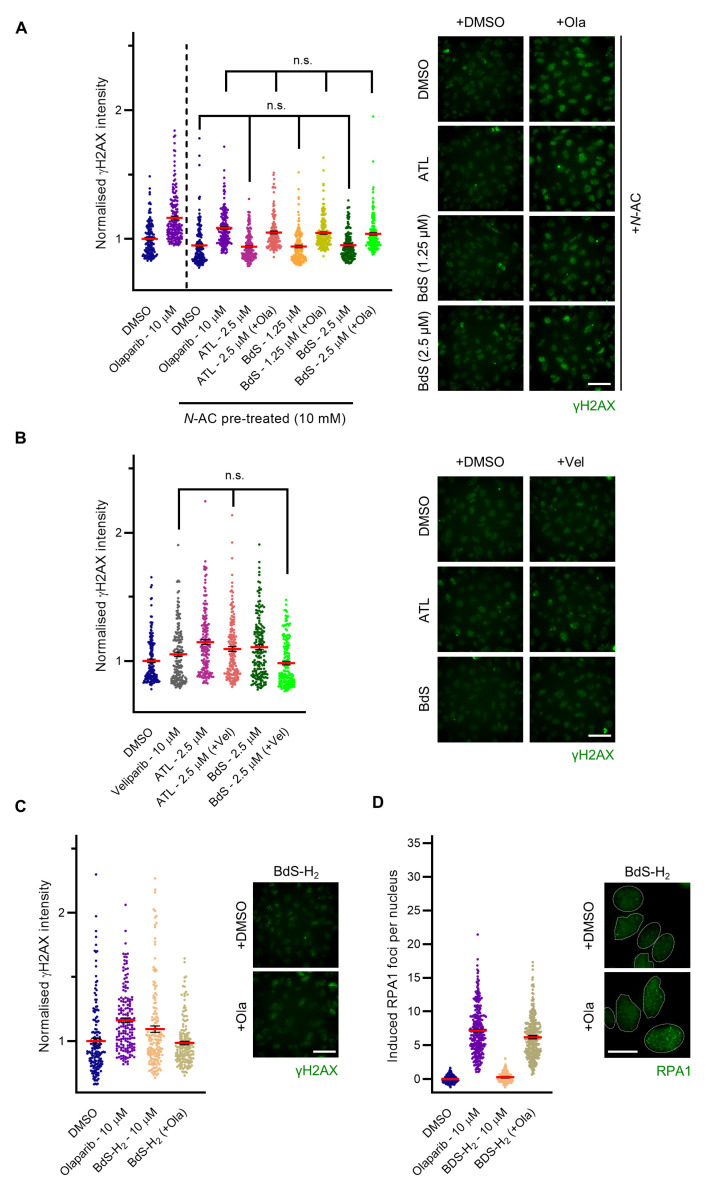
Cooperative effects of BdS and alantolactone (ATL) with olaparib depend on oxidative stress, PARP-trapping, and covalent binding capacity. (**A**) Quantification (left) and representative images (right) of normalised nuclear γH2AX immunofluorescence intensity (Alexa Fluor 488) of ≥ 7500 U2OS cells (3 replicates; *n* = 168) following 24 h treatment with olaparib, BdS, and/or ATL as indicated, compared to vehicle only (DMSO) after *N*-acetylcysteine (*N*-AC) pre-treatment (10 mM, 1 h). Data points relate to each recorded field (mean ± SEM). Scale bar—50 μm. (**B**) Quantification (left) and representative images (right) of normalised nuclear γH2AX immunofluorescence intensity (Alexa Fluor 488) of ≥ 7500 U2OS cells (3 replicates; *n* = 168) following 24 h treatment with veliparib, BdS, and/or ATL as indicated, compared to vehicle only (DMSO). Data points relate to each recorded field (mean ± SEM). Scale bar—50 μm. (**C**) Quantification (left) and representative images (right) of normalised nuclear γH2AX immunofluorescence intensity (Alexa Fluor 488) of ≥ 7500 U2OS cells (3 replicates; *n* = 168) following 24 h treatment with olaparib and/or BdS-H_2_ as indicated, compared to vehicle only (DMSO). Data points relate to each recorded field (mean ± SEM). Scale bar—50 μm. (**D**) Quantification (left) and representative images (right) of induced RPA1 foci per cell (Alexa Fluor 488) of ≥ 18,000 U2OS cells (2 independent experiments; *n* = 336) following 24 h treatment with olaparib and/or BdS-H_2_ as indicated, compared to vehicle only (DMSO). Data points relate to each recorded field (mean ± SEM). DMSO and olaparib controls are identical to the ones displayed in Figure 3B due to forming part of the same experimental pipeline. Nuclei are outlined in white using DAPI as a reference. Scale bar—20 μm. Statistical significance indicated as follows: n.s.—*p* > 0.05.

To clarify the nature of the DNA damage potentiation, we incorporated *N*-AC into the experimental pipeline, which is a biological precursor of glutathione and an antioxidant [14]. As such, pre-treatment of cells with *N*-AC is expected to attenuate ROS-mediated contributions to combination treatments, thereby providing insights into the nature of the observed phenotypes. Indeed, pre-treatment with *N*-AC (10 mM, 1 h) prevented significant DNA damage potentiation in the combination treatments (Figure 4A). Instead, the resulting damage was equivalent to that of olaparib treatment alone, presumably as a consequence of stochastic replication fork collisions with PARP–DNA complexes [5,7]. The effects of basal ROS levels on this type of damage were minor (apparent when comparing olaparib-treated populations in the absence and presence of *N*-AC pre-treatment in Figure 4A).

Secondly, to test if the potentiation of DNA damage was due to an increase in frequency of trapped PARP–DNA complexes, we compared the effects of olaparib with those of veliparib (10 μM), a small molecule, which inhibits PARP enzyme function to a similar extent to olaparib but displays far less trapping activity [7,8]. Compared to olaparib, combination treatments of BdS/ATL with veliparib resulted in no statistically significant changes in γH2AX intensity compared to veliparib alone (Figure 4B), indicating that the observed effects were likely due to PARP trapping rather than enzymatic PARP inhibition.

Finally, we combined the inert BdS analogue, BdS-H_2_, with olaparib. Even at a four-fold higher dose (10 μM) than the highest assay concentration of BdS, BdS-H_2_ was unable to potentiate the γH2AX induction of olaparib (compare Figure 4C with Figure 3A, noting y-axis scaling). Similarly, the addition of BdS-H_2_ to olaparib did not recapitulate the synergistic RPA1 foci increase we had observed for BdS and olaparib (compare Figure 4D with Figure 3B). Taken together, these findings demonstrate that the compound’s Michael acceptor was required for the enhanced DNA damage induction, highlighting the importance of BdS’s electrophilicity and covalent binding character to the DNA damage phenotypes studied.

### 2.6. Combination Treatment of BdS or ATL with Olaparib Induces Pleiotropic Mitotic Defects

**Figure 5:**
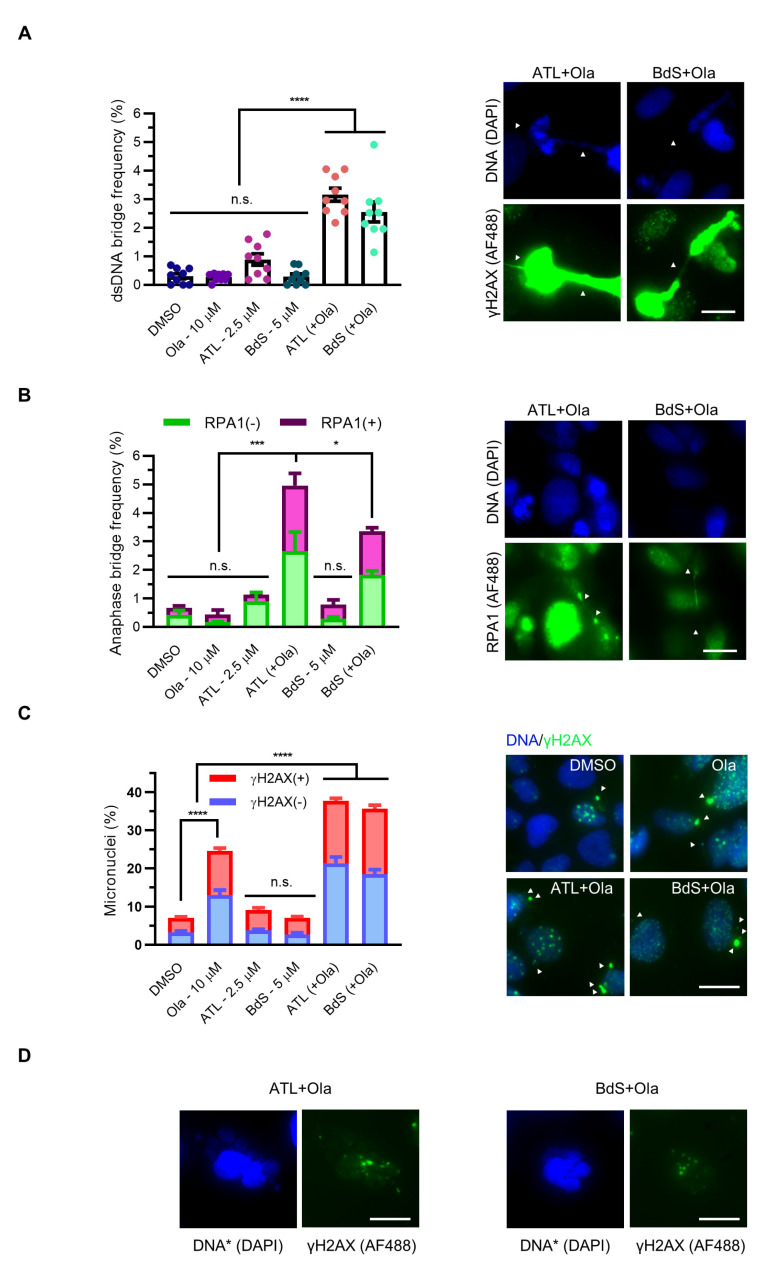
BdS and alantolactone (ATL) synergistically potentiate olaparib-induced mitotic defects in p53 wildtype cancer cells. (**A**) Quantification (left) and representative images (right, Alexa Fluor 488; AF488) of percentages of U2OS cells displaying bulky anaphase bridges (DAPI-positive) after 72 h treatment with the specified compounds alone or in combination as indicated compared to vehicle only (DMSO). Each dataset represents a minimum of 4500 U2OS cells (3 independent experiments; *n* = 9; mean ± SEM). White arrowheads indicate anaphase bridges. Scale bar—20 μm. (**B**) Quantification (left) and representative images (right, Alexa Fluor 488; AF488) of percentages of U2OS cells displaying ultra-fine anaphase bridges (DAPI-negative, RPA1-positive; shown in magenta bars) in addition to bulky bridges (shown in green bars) after 72 h treatment with the specified compounds alone or in combination as indicated compared to DMSO. Each dataset represents a minimum of 1500 U2OS cells (3 replicates; *n* = 3; mean ± SEM). White arrowheads indicate opposite bridge ends for the two combination treatments. Scale bar—20 μm. (**C**) Quantification (left) and representative images (right, Alexa Fluor 488 and DAPI merge) of percentages of U2OS cells displaying γH2AX-negative (−) and -positive (+) micronuclei after 72 h treatment with the specified compounds alone or in combination as indicated compared to vehicle only (DMSO). Each dataset represents a minimum of 4500 U2OS cells (3 independent experiments; *n* = 9; mean ± SEM). Micronuclei are indicated using white arrowheads. Scale bar—20 μm. (**D**) Representative fluorescent images of multinucleated cells, with their DNA (DAPI; * high-exposure) and DNA damage (anti-γH2AX; Alexa Fluor 488) stained. Scale bar—20 μm. Statistical significance indicated as follows: n.s.—*p* > 0.05, *—*p* < 0.05, ***—*p* < 0.001, and ****—*p* < 0.0001.

Given the established mitotic effects of both olaparib and increased basal ROS levels, we investigated whether combination treatments of BdS or ATL with olaparib could stress mitotic fidelity [7]. We observed numerous mitotic defects, providing us with an improved understanding of the cellular sequelae induced by the combined drug treatments. Notably, BdS or ATL in combination with olaparib resulted in highly synergistic increases in bulky chromatin bridge formation (Figure 5A). Additional bridges were identified following RPA1 staining, representing the induction of two key anaphase bridge subtypes by the combination treatments: bulky, DAPI-positive chromatin bridges and ultra-fine, DAPI-negative but RPA1-positive (RPA1+) bridges (Figure 5B; both subtypes represented) [49,50]. In line with mitotic dysregulation, we also detected a synergistic increase in the percentage of micronuclei present upon combination treatment of BdS or ATL with olaparib (Figure 5C). The micronuclei could be subdivided into two distinct populations that were either positive or negative for γH2AX staining [51,52]. These aberrations can be indicative of chromosome mis-segregation errors, possibly as a result of under-replicated regions of DNA entering into mitosis. Alternatively, they can be due to microtubule stabilisation induced by mild replication stress preventing successful cytokinesis [5,7,9,53,54]. Following aberrant mitoses, it is foreseeable that some cells would enter senescence or controlled apoptosis. Consistent with this idea, we observed the presence of various multinucleated cells indicative of quiescence and/or apoptosis [55]. The multinucleated cells were detectable to varying extents, and particularly in the cells treated with BdS/ATL in combination with olaparib (representative images in Figure 5D).

### 2.7. BdS or ATL Combination Treatment with Olaparib Results in G2 Cell-Cycle Stalling

**Figure 6:**
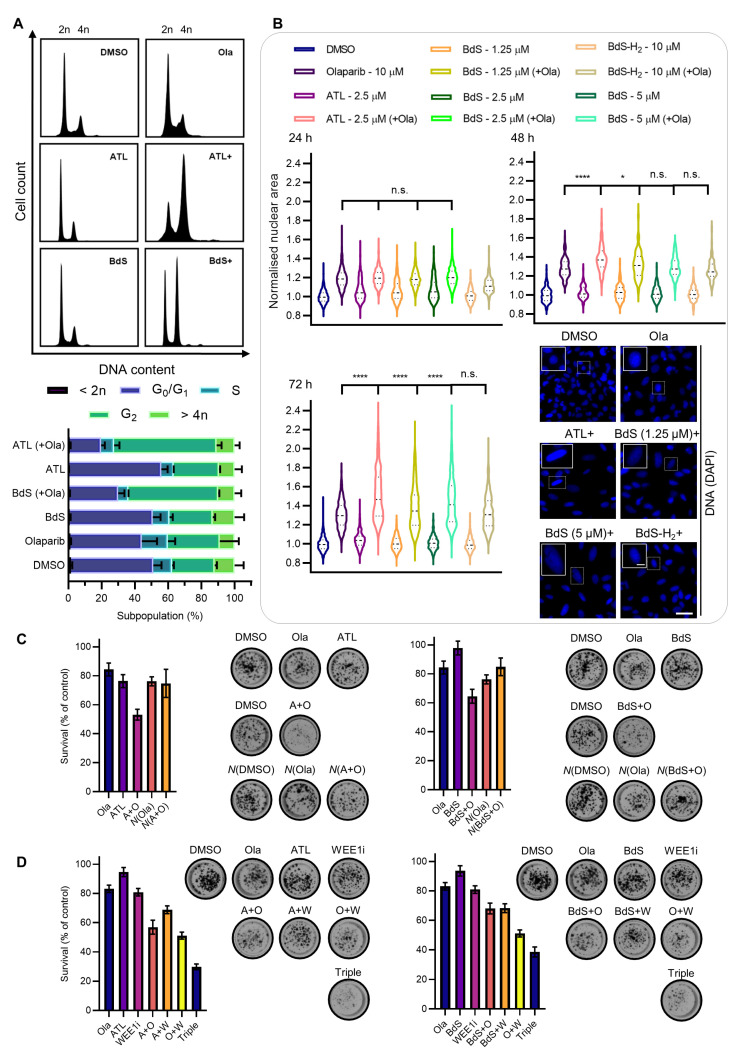
BdS and alantolactone (ATL) synergistically potentiate olaparib-induced cell growth inhibition in p53 wildtype cancer cells. (**A**) Propidium iodide (PI)-treated U2OS cell-cycle analysis after 48 h of drug treatment. Representative cell-cycle distributions depicting DNA content (top) and histograms (bottom) are shown for two independent experiments (*n* = 2; mean ± SEM). (**B**) Normalised nuclear area quantifications and representative images of the DAPI-stained U2OS nuclei (bottom right; 72 h time point) after treatment with olaparib (10 μM), ATL (2.5 μM), BdS (1.25, 2.5, 5 μM) and/or BdS-H_2_ (10 μM) at the indicated time points (dotted lines show median and quartiles). For the 24 h time point, each dataset represents a minimum of 25,000 cells (4 independent experiments; *n* = 672, except for BdS-H_2_ and its combination treatment which represent a minimum of 6250 cells—1 independent experiment; *n* = 168). For the 48 h time point, each dataset represents a minimum of 7500 cells (1 independent experiment; *n* = 168). For the 72 h time point, each dataset represents a minimum of 15,000 cells (3 independent experiments; *n* = 504). Scale bars—20 and 5 μm in inset. (**C**) Clonogenic survival assays with representative, stained colony images. Chronic treatment of U2OS cells (≥3 independent experiments; *n* ≥ 3; mean ± SEM) with alantolactone (ATL or A; 0.5 μM, left) and BdS (5 μM, right) in combination with olaparib (Ola or O; 0.625 μM). (**D**) Clonogenic survival assays with representative, stained colony images. Chronic treatment of U2OS cells (3 independent experiments; *n* ≥ 6; mean ± SEM) as in (**C**) (olaparib; 0.625 μM, BdS; 3.75 μM, ATL; 0.375 μM) but in combination with AZD1775, a WEE1 inhibitor (WEE1i or W; 0.05 μM). Controls for ATL and BdS are identical due to forming part of the same experimental pipeline (Ola, WEE1i, O + W). Statistical significance indicated as follows: n.s.—*p* > 0.05, *—*p* < 0.05, and ****—*p* < 0.0001.

From preceding assays, it was clear that DNA damage, inflicted by the above drug combinations, coincided with significant increases in mitotic errors. Given these elements, we looked at cell-cycle progression at the time point immediately preceding the culmination of this phenotype (48 h) seeking further mechanistic insights (Figure 6A). Treatment with ATL or BdS (each at 10 μM) resulted in negligible effects on U2OS cell-cycle progression, besides a slim increase in the G1 subpopulation following ATL administration, suggesting that significant replication stress is only incurred by combination treatments once extensive PARP-trapping becomes an obstacle to DNA replication.

For olaparib treatment alone, we observed some G2 stalling consistent with previous research [25], which was accompanied by minor S phase subpopulation growth. Most strikingly however, upon combining ATL or BdS with olaparib, a profound increase in the G2 population was observed, consistent with previous findings for ATL [14], and a significant rise in nuclear area developed in olaparib-treated cells at 72 h (Figure 6B). The average nuclear size increase was further exacerbated by the inclusion of ATL or BdS. By contrast, in cells treated with BdS-H_2_ it remained comparable to that of olaparib treatment alone, indicating that BdS’s electrophilicity was the source of this biological activity, which is consistent with preceding assays.

### 2.8. BdS/ATL-Enhanced Olaparib-Mediated Cell Death in p53 wt Cancer Cells Is Further Potentiated by WEE1 Inhibition

To see if the DNA damage, mitotic aberrations, and cell-cycle alterations induced by ATL/BdS and olaparib combination treatments would manifest as longer-term survival defects, we performed clonogenic survival assays in U2OS cells that were chronically exposed to ATL/BdS-olaparib combination treatments. The clear, mildly synergistic potentiation of cytotoxicity arising from the drug combination was rescued to the level of growth inhibition of olaparib alone through pre-treatment of the cells with *N*-AC (Figure 6C), demonstrating that the potentiation of olaparib-mediated growth inhibition is likely ROS-dependent. As expected, the combination of olaparib with WEE1 inhibition was synergistic, which is consistent with synergy studies in p53-deficient environments [56]. However, the addition of BdS or ATL to this combination treatment further potentiated the observed cell toxicity and markedly reduced the size of the individual colonies (Figure 6D), demonstrating the cooperative nature of the respective drug combinations.

## 3. Discussion

In this work, we establish that in a p53 wildtype setting the combination of olaparib-mediated PARP-trapping and ATL/BdS-mediated ROS accumulation leads to potentiation of DNA damage and mitotic defects caused by olaparib alone. The biological activity of both ATL and BdS can be assigned to their shared electrophilic α-methylene-γ-butyrolactone warhead, while the PARP-trapping function of olaparib is essential for the observed phenotypes. While BdS was able to attenuate the ubiquitin-loading of UBE2D enzymes in vitro, its inability to inhibit UBE2Ds or affect DNA repair in a range of cancer cell lines point towards the engagement of other endogenous cellular nucleophiles by the compound. A potential target is TrxR, which exhibits selenocysteine catalytic residues that rarely feature in the human proteome (~20 proteins, often buried) and have a significantly lower pK_a_ (as a selenolate) than other endogenous nucleophiles, such as cysteines (as a thiolate) [57].

The development of moderately electrophilic small molecules targeting TrxR via covalent inhibition could therefore represent an interesting therapeutic modality to extend the usage of olaparib and other PARP-trapping inhibitors in the future [57,58,59]. Meanwhile, the inhibition of UBE2D enzymes and/or other factors to impede CtIP function remains an untapped and viable research avenue, as evidenced by recent work [60].

Combining chronic olaparib treatment with either ATL or BdS allowed us to shed light on the nature of the DNA damage and mitotic defects caused by the respective cellular effects they induce [14]. Varying perturbations point to a replication stress-driven mitotic phenotype that develops following progressive RPA pool depletion, leading to a profound G2 arrest, likely p53-mediated, which is protective to cell fate. This arrest can be overcome by abrogating the G2/M checkpoint via WEE1 inhibition, which further reduced clonogenic survival.

At 72 h, combination treatments induced synergistic levels of DNA damage, and a significant number of mitotic defects, associated with chromosome mis-segregation [61]. The destabilisation of replication forks via physical impediment, such as PARP-trapping, typically culminates in mitotic catastrophe, producing multinucleation or macronucleation, or micronucleation in the case of acentric or lagging chromosomes, as seen in this work [7,25,62]. As a result, the micronuclei consisted of both the γH2AX(+) subtype, arising from DSB clusters occurring in S phase and the γH2AX(−) subtype, typically resulting from lagging chromosomes [51,52]. The chromatin bridging we observed characteristically occurs at replication intermediates, such as stalled replication forks or under-replicated regions (e.g., common fragile sites) that enter mitosis despite their intermediate state, or dicentric chromosomes more generally [5,7,49,50,62]. Such events have been observed following transient G2 delays due to mild replication stress, which is consistent with the sequence of events we observed [54]. In sum, the aberrations we detected that are substantially exacerbated by BdS or ATL treatment are consistent with PARP-trapping and most likely via ROS-induced DNA damage. The precise causes and order of events for these defects require further investigation.

Given the increasing potential for early cancer diagnoses, it is apt to consider tailored treatment options for these early-stage cancers, which are often wildtype for p53. In fact, ~50% of all detected cancers are thought to retain p53 functionality [26]. Taken together, this study provides insights into how these cancers, particularly those with high levels of basal oxidative stress, might be targeted in the future by extending the use of PARP-trapping drugs via the exploitation of ROS-potentiated replication stress [63].

## 4. Materials and Methods

### 4.1. Chemical Synthesis

Commercial *n*-butyl lithium in hexanes was titrated using L-menthol crystals and 1,10-phenanthroline prior to use. Diisopropylamine was distilled over calcium hydride prior to use. Lithium diisopropylamide was prepared from freshly distilled diisopropylamine and titrated *n*-butyl lithium. All other solvents and reagents were purchased from Acros (Fisher; Geel, Belgium), Aldrich (Merck; St. Louis, MO, USA), Alfa Aesar (Haverhill, MA, USA) and Fluorochem (Hadfield, UK), and used without further purification. All air and moisture-sensitive reactions were performed under a nitrogen atmosphere in oven-dried and flame-dried glassware. Thin layer chromatography was performed using pre-coated silica gel F_254_ plates. The plates were viewed under UV light or using an aqueous basic potassium permanganate (KMnO_4_) solution. Column chromatography was carried out using flash techniques on silica gel (particle size 40–63 μm). NMR data were collected on a Bruker (Billerica, MA, USA) Avance III 400 MHz or Bruker Avance II+ 500 MHz spectrometers. Chemical shifts are given in ppm (δ) and are referenced to the residual CDCl_3_ solvent peak at 7.26 ppm (^1^H NMR) and 77.16 ppm (^13^C NMR) or to the residual CH_3_OD solvent peak at 3.31 (^1^H NMR), and quoted in ppm to 2 decimal places with coupling constants (J) to the nearest 0.1 Hz. Other abbreviations used are s (singlet), d (doublet), t (triplet), m (multiplet), and br (broad). High-resolution mass spectra (HRMS) were performed by the School of Chemistry Mass Spectrometry Service (University of Manchester) employing a Thermo Finnigan MAT95XP spectrometer.

**(3R,3aR,5aS,9bS)-3,5a,9-Trimethyl-3-(phenylselenyl)3a,5,5a,9btetrahydronaphtho[1,2-b]furan-2,8(3H,4H)-dione (2)**

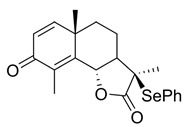


To a stirred −78 °C solution of (−)-α-santonin (492.6 mg, 2 mmol) in dry THF (7.6 mL) under nitrogen was added dropwise LDA (2 M, 1.2 equiv) via syringe. After 30 min, a solution of PhSeCl (1.1 equiv) in dry THF (3.3 mL) was added dropwise via syringe to the above mixture while keeping the temperature within 5 °C of −78 °C. The reaction solution was stirred at −78 °C for 1 h and then at room temperature (RT) overnight. The reaction was detected complete by TLC and then quenched by the addition of the saturated aqueous NH_4_Cl. The mixture was extracted with EtOAc (3 × 10 mL) and washed with brine (1 × 20 mL). The combined organic layers were dried over anh. MgSO_4_ and evaporated under vacuum to give a yellow residue that was purified by silica-gel column chromatography (EtOAc/petroleum ether: 4–20%), resulting in a white solid product (369.3 mg, 46%). 

^1^H NMR (500 MHz, CDCl_3_) δ 7.64 (dd, *J* = 8.0, 1.4 Hz, 2H), 7.49-7.40 (m, 1H), 7.35 (t, *J* = 7.6 Hz, 2H), 6.68 (d, *J* = 9.9 Hz, 1H), 6.24 (d, *J* = 9.9 Hz, 1H), 5.26-5.17 (m, 1H), 2.13 (d, *J* = 1.3 Hz, 3H), 2.01-1.89 (m, 4H), 1.60 (s, 3H), 1.54-1.47 (m, 1H), 1.32 (s, 3H). ^13^C NMR (126 MHz, CDCl_3_) δ 186.0, 174.6, 154.6, 150.8, 138.1, 129.9, 129.1, 125.9, 123.8, 79.2, 57.4, 48.7, 41.2, 37.4, 24.9, 22.2, 20.4, 10.9. These data were consistent with those published [21].

**(3aS,5aS,9bS)-5a,9-Dimethyl-3-methylene-3a,5,5a,9b-tetrahydronaphtho[1,2-b]furan-2,8(3H,4H)-dione (3)**

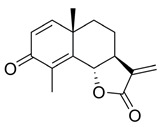


To a solution of **2** (220.7 mg, 0.55 mmol) and AcOH (3.0 equiv) in THF (1.46 mL) at 0 °C was added dropwise 30% H_2_O_2_ (6.5 equiv). The reaction solution was stirred at 0 °C for 20 min and then at RT for 10 min, under nitrogen. The reaction was quenched by the addition of saturated aqueous NaHCO_3_ (5 mL). The mixture was extracted with EtOAc (3 × 5 mL) and washed with brine (1 × 15 mL). The combined organic layers were dried over anh. MgSO4 and evaporated under vacuum to give a colourless residue that was purified by silica-gel column chromatography (EtOAc/hexane: 10–50%), resulting in a white solid product (95.0 mg, 71%).

^1^H NMR (500 MHz, CDCl_3_) δ 6.70 (d, *J* = 9.9 Hz, 1H), 6.28-6.20 (m, 2H), 5.56 (s, 1H), 4.76 (d, *J* = 11.6 Hz, 1H), 2.75-2.65 (m, 1H), 2.24-2.17 (m, 1H), 2.15 (s, 3H), 1.92 (d, *J* = 13.5 Hz, 1H), 1.83-1.70 (m, 1H), 1.63-1.55 (m, 1H), 1.31 (s, 3H). ^13^C NMR (126 MHz, CDCl_3_) δ 186.3, 169.2, 154.8, 150.8, 137.6, 129.1, 126.1, 119.8, 81.6, 50.4, 41.5, 37.8, 25.3, 21.8, 11.0. These data were consistent with those published [21].

**(3aS,9bR)-6,9-Dimethyl-3-methylene-2-oxo-2,3,3a,4,5,9b-hexahydronaphtho[1,2-b]furan-8-yl acetate (4)**

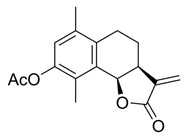


To a solution of **3** (75.7 mg, 0.31 mmol) in Ac_2_O (0.38 mL) being cooled to 0 °C was added dropwise conc. H_2_SO_4_ (two drops—~10 µL). The reaction mixture was stirred for 2.75 h and then was quenched with the addition of ice (5 g). A solution of NaOH (1 M) was added until neutral pH was detected. It was then extracted with dichloromethane (3 × 10 mL) and washed with sat. aq. NaHCO_3_ and brine (1 × 20 mL each). The combined organic layers were dried over anh. MgSO_4_ and evaporated under vacuum to give crude product that was purified by silica-gel column chromatography (EtOAc/hexane: 15-60%), resulting in a white solid product (68.4 mg, 77%).

^1^H NMR (400 MHz, CDCl_3_) δ 6.87 (s, 1H), 6.31 (s, 1H), 5.72 (s, 1H), 5.56 (s, 1H), 3.34-3.27 (m, 1H), 2.74 (dt, *J* = 16.7, 4.7 Hz, 1H), 2.53 (ddd, *J* = 17.0, 9.7, 4.7 Hz, 1H), 2.33 (s, 3H), 2.23 (s, 3H), 1.98 (dd, *J* = 13.5, 6.1 Hz, 1H), 1.81 (dtd, *J* = 14.0, 9.7, 4.5 Hz, 1H). ^13^C NMR (101 MHz, CDCl_3_) δ 170.2, 169.8, 147.5, 139.8, 134.9, 134.6, 131.1, 129.1, 124.1, 121.8, 74.9, 39.4, 25.9, 24.1, 20.9, 19.7, 12.1. These data were consistent with those published [21].

**(3aS,9bR)-8-Hydroxy-6,9-dimethyl-3-methylene-3a,4,5,9btetrahydronaphtho[1,2-b]furan-2(3H)-one (5)**

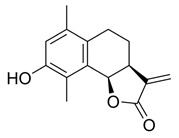


To a solution of **4** (68.7 mg, 0.24 mmol) in methanol (0.79 mL) being cooled to 0 °C was added dropwise ammonium hydroxide (35.6%, 0.79 mL). The reaction was stirred for 7 h, then the mixture was concentrated under vacuum briefly and extracted with EtOAc (3 × 5 mL), then washed with brine (1 × 15 mL). The combined organic layers were dried over anh. MgSO_4_ and evaporated under vacuum to give a colourless residue that was purified by silica-gel column chromatography (EtOAc/hexane: 20–60%), resulting in a yellow solid product (28.5 mg, 49%).

^1^H NMR (500 MHz, CDCl_3_) δ 6.68 (s, 1H), 6.30 (s, 1H), 5.71 (s, 1H), 5.59 (d, *J* = 6.7 Hz, 1H), 3.33-3.27 (m, 1H), 2.69 (dd, *J* = 11.7, 5.9 Hz, 1H), 2.52-2.46 (m, 1H), 2.30 (s, 3H), 2.18 (s, 3H), 1.96 (dt, *J* = 8.7, 6.0 Hz, 1H), 1.79 (dd, *J* = 15.8, 7.4 Hz, 1H). ^13^C NMR (126 MHz, CDCl_3_) δ 170.5, 152.0, 140.1, 134.5, 130.7, 128.7, 122.6, 121.6, 117.9, 75.3, 39.6, 23.7, 19.7, 11.4. These data were consistent with those published [21].

**(3aS,9bR)-8-((2-Bromobenzyl)oxy)-6,9-dimethyl-3-methylene3a,4,5,9b-tetrahydronaphtho[1,2-b] furan-2(3H)-one (BdS, 6)**

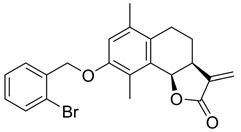


To a solution of **5** (7.3 mg, 0.03 mmol) and 2-bromobenzyl bromide (2.0 equiv) in acetone (1.35 mL) was added K_2_CO_3_ (1.1 equiv). The reaction suspension was stirred at ambient temperature overnight under nitrogen. Then, the mixture was filtered through a celite pad, and the filtrate was concentrated to give the crude product, which was purified by preparative thin layer chromatography (EtOAc/petroleum ether: 16.6%), resulting in a yellow solid product (8.1 mg, 65%).

^1^H NMR (500 MHz, CDCl_3_) δ 7.59 (d, *J* = 7.9 Hz, 2H), 7.37 (d, *J* = 7.5 Hz, 1H), 7.20 (t, *J* = 7.5 Hz, 1H), 6.80 (s, 1H), 6.31 (s, 1H), 5.72 (s, 1H), 5.64 (d, *J* = 6.7 Hz, 1H), 5.11 (s, 2H), 3.34-3.29 (m, 1H), 2.76-2.70 (m, 1H), 2.54-2.49 (m, 1H), 2.40 (s, 3H), 2.24 (s, 3H), 1.96 (dd, *J* = 13.4, 4.7 Hz, 1H), 1.82 (dd, *J* = 11.4, 6.4 Hz, 1H). ^13^C NMR (126 MHz, CDCl_3_) δ 170.4, 154.7, 140.2, 136.8, 134.3, 132.7, 130.8, 129.3, 129.0, 128.8, 127.8, 126.2, 122.3, 121.5, 115.1, 75.2, 70.0, 39.6, 29.9, 26.3, 23.8, 20.2, 11.8. These data were consistent with those published [21].

**(3S,3aS,9bR)-3,6,9-Trimethyl-2-oxo-2,3,3a,4,5,9b-hexahydronaphtho[1,2-b]furan-8-yl acetate (7)**

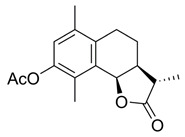


According to the synthetic method for the preparation of **4** using (−)-α-santonin as starting material (150 mg, 0.61 mmol), a white solid was obtained (140.6 mg, 83%).

^1^H NMR (400 MHz, CDCl_3_) δ 6.86 (s, 1H), 5.59 (s, 1H), 2.74 (dt, *J* = 17.2, 5.5 Hz, 1H), 2.59-2.48 (m, 2H), 2.42 (dq, *J* = 9.6, 4.7 Hz, 1H), 2.32 (s, 3H), 2.21 (d, *J* = 2.4 Hz, 6H), 1.93 (dt, *J* = 10.7, 5.4 Hz, 1H), 1.79-1.68 (m, 1H), 1.39 (d, *J* = 7.4 Hz, 3H). ^13^C NMR (126 MHz, CDCl_3_) δ 179.4, 169.7, 147.5, 134.9, 134.2, 131.4, 129.1, 124.0, 75.6, 41.7, 40.5, 24.1, 23.5, 20.9, 19.7, 14.6, 12.2. These data were consistent with those published [64].

**(3S,3aS,9bR)-8-Hydroxy-3,6,9-trimethyl-3a,4,5,9b-tetrahydronaphtho[1,2-b]furan-2(3H)-one (8)**

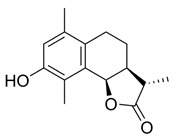


According to synthetic method for the preparation of **5** using **7** as starting material (100 mg, 0.35 mmol), a white solid was obtained (79.6 mg, 92%).

^1^H NMR (500 MHz, MeOD) δ 6.66 (s, 1H), 5.72 (d, *J* = 6.1 Hz, 1H), 2.77-2.67 (m, 1H), 2.58-2.52 (m, 1H), 2.52-2.41 (m, 2H), 2.21 (s, 3H), 2.16 (s, 3H), 1.92 (dd, *J* = 12.0, 7.4 Hz, 1H), 1.67-1.59 (m, 1H), 1.37 (d, *J* = 7.4 Hz, 3H). ^13^C NMR (126 MHz, CDCl_3_) δ 179.8, 152.0, 134.7, 131.1, 128.4, 122.5, 117.7, 76.0, 42.0, 40.5, 23.7, 19.7, 14.6, 11.6. These data were consistent with those published [64].

**(3S,3aS,9bR)-8-((2-Bromobenzyl)oxy)-3,6,9-trimethyl-3a,4,5,9b-tetrahydronaphtho[1,2-b]furan-2(3H)-one (BdS-H_2_, 9)**

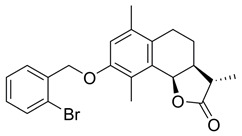


According to the synthetic method for the preparation of **6** using **8** as starting material (17.2 mg, 0.07 mmol). Purification via column chromatography (EtOAc/hexane: 10-35%) yielded a white solid product (6.9 mg, 25%).

^1^H NMR (400 MHz, CDCl_3_) δ 7.59 (d, *J* = 8.0 Hz, 2H), 7.36 (t, *J* = 7.5 Hz, 1H), 7.19 (t, *J* = 7.6 Hz, 1H), 6.79 (s, 1H), 5.65 (d, *J* = 6.3 Hz, 1H), 5.10 (s, 2H), 2.80-2.65 (m, 1H), 2.60-2.47 (m, 2H), 2.43 (dd, *J* = 10.7, 5.0 Hz, 1H), 2.37 (s, 3H), 2.24 (s, 3H), 1.93 (dd, *J* = 13.5, 6.1 Hz, 1H), 1.79-1.70 (m, 1H), 1.40 (d, *J* = 7.4 Hz, 3H). ^13^C NMR (101 MHz, CDCl_3_) δ 179.4, 154.4, 136.5, 134.1, 132.4, 130.8, 128.9, 128.5, 128.3, 127.4, 125.9, 121.9, 114.6, 75.6, 69.7, 41.7, 40.3, 23.5, 19.8, 14.4, 11.7. HRMS (HESI): *m*/*z* calcd for C_22_H_23_O_3_Br [M + Na]^+^ 437.0723, found 437.0713.

### 4.2. Biology

**Commercial compounds used in biological assays.** Stock solutions for alantolactone (ATL; cat#SML0415; Sigma Aldrich; St. Louis, MO, USA), olaparib (cat#A10111-10; Generon; Houston, Texas, United States), veliparib (cat#HY-10129-5mg; Cambridge Bioscience; Cambridge, UK), MG-132 (cat#474787-10MG; VWR international; Radnor, Pennsylvania, United States), and bortezomib (cat#A10160-10; Generon; Houston, Texas, United States) were prepared at 100 mM in dimethyl sulfoxide (DMSO; Sigma Aldrich; St. Louis, MO, USA), for PYR-41 (cat#N2915; Sigma Aldrich; St. Louis, MO, USA) at 50 mM in DMSO, for MK-1775 (cat#21266; Cayman Chemicals; Ann Arbor, MI, USA) at 10 mM in DMSO, and for 4’,6’-Diamidino-2-phenylindole dihydrochloride (DAPI; Acros organics; Geel, Belgium) at 1 mg mL^−1^ stock in DMSO. *N*-acetylcysteine (*N*-AC; cat#A9165-5G; Merck Life Science; Darmstadt, Germany) was filter purified (0.22 μm) and used as a 10 mM stock in cell growth medium, as was tert-butyl hydroperoxide (t-BHP; cat#A13926.AE; Alfa Aesar; Haverhill, MA, USA).

**In vitro ubiquitin-loading assays.** In vitro ubiquitin-loading assays were carried out in assay buffer (25 mM Tris-HCl pH 7.5, 50 mM MgCl_2_). Recombinant UBE2D1 or UBE2D3 (cat#E2-616-100, cat#E2-627-100; Boston Biochem; Cambridge, MA, USA; 0.5 μM) were pre-incubated with the requisite concentration of drug (21 °C, 15 min.). Then, ATP (Sigma Aldrich; St. Louis, MO, USA; 2 mM), His_6_-UBA1 (cat#E-304-050; Boston Biochem; Cambridge, MA, USA; 0.2 μM), and mono-ubiquitin (10 μM) were added. Subsequently, the reactions were incubated (37 °C, 40 min) using a thermal cycler (Fisher Science; Waltham, MA, USA; Mini Amp Plus). The reactions were terminated with non-reducing 2x SDS PAGE sample buffer (resulting 1×—10% glycerol, 60 mM Tris HCl pH 6.8, 2% SDS, 0.01% bromophenol blue) and heated at 95 °C for 3 min prior to gel loading.

**Cell culture.** All cell lines were cultured at 37 °C in a humidified, 5% CO_2_ atmosphere. U2OS (RRID: CVCL_0042) and U2OS-derived cells were grown in high-glucose Dulbecco’s Modified Eagle Medium (DMEM; Sigma Aldrich; St. Louis, MO, USA; cat#D6546) supplemented with 10% (*v*/*v*) foetal bovine serum (FBS; Sigma Aldrich; St. Louis, MO, USA; cat#10270106), 100 U mL^−1^ penicillin (Gibco; Waltham, MA, USA), 100 μg mL^−1^ streptomycin (Gibco; Waltham, MA, USA), and 2 mM glutamine (Gibco; Waltham, MA, USA). Additional supplements were added to the following stable cell lines: 2 μg mL^−1^ blasticidin (Invitrogen; Waltham, MA, USA) and 0.5 mg mL^−1^ G418 (Gibco; Waltham, MA, USA; cat#10131035) for U2OS Trex cells stably expressing inducible siALL-Ds-resistant GFP-UBE2D1, wt, and C85S; and 2 μg mL^−1^ blasticidin (Invitrogen; Waltham, MA, USA) and 0.2 mg mL^−1^ zeocin (Invitrogen; Waltham, MA, USA; cat#R25001) for U2OS Trex cells stably expressing inducible GFP, as described previously [12]. For routine induction, doxycycline (Fisher Scientific; Waltham, MA, USA) was added at final concentrations of 0.002, 0.009, or 12 µg ml^−1^ for 24 h to induce comparable levels of GFP, GFP-UBE2D1 CD, or GFP-UBE2D1 wt expression, respectively. OVCAR3 and Kuramochi cell lines stably expressing GFP-tagged histone H2B were kindly provided by the Taylor lab [34,36] and grown in Roswell Park Memorial Institute (RPMI) 1640 Medium, supplemented with glutamine (Gibco; Waltham, MA, USA; cat#21875034), 10% (*v*/*v*) foetal bovine serum (FBS; Sigma Aldrich; St. Louis, MO, USA), 100 U mL^−1^ penicillin (Gibco; Waltham, MA, USA), 100 μg mL^−1^ streptomycin (Gibco; Waltham, MA, USA), and an additional 2 mM glutamine (Gibco; Waltham, MA, USA). GFP-H2B COV318 cells [34,36] were grown in high-glucose Dulbecco’s Modified Eagle Medium (DMEM; Sigma Aldrich; St. Louis, MO, USA) supplemented with 10% (*v*/*v*) foetal bovine serum (FBS; Sigma Aldrich; St. Louis, MO, USA; cat#10270106), 100 U mL^−1^ penicillin (Gibco; Waltham, MA, USA), 100 μg mL^−1^ streptomycin (Gibco; Waltham, MA, USA), and 2 mM glutamine (Gibco; Waltham, MA, USA). All cell lines were authenticated prior to use and routinely tested for mycoplasma. Cells used experimentally were passaged a minimum of twice and a maximum of ten times prior to use.

**Cellular auto-ubiquitylation assays.** U2OS-derived cells were seeded in 60 mm dishes and transfected with the requisite siRNAs on two consecutive days, following the manufacturer’s guidelines (Lipofectamine RNAiMAX; Invitrogen; Waltham, MA, USA). A total of 48 h after the first transfection, the cells were induced using doxycycline (24 h). Following the indicated drug treatment, cells were harvested in lysis buffer (Tris/HCl 50 mM—pH 7.5, 2% SDS, 10 mM N-ethylmaleimide, 1x cOmplete protease, and 1x phosphatase inhibitors). After heating for 1 min at 95 °C, samples were syringed through a 25 G needle and cleared by centrifuging at 17,000 g for 5 min. The protein content in the supernatants of all samples was normalised, reducing SDS sample buffer added to 1x concentration as above and heated at 95 °C for 3 min before gel loading for SDS PAGE.

**siRNA.** The sequences of siRNAs used in this study are as described in previous work, with siALL-Ds—an equimolar mixture (1:1:1) of siUBE2D1-2, siUBE2D2-1 and siUBE2D4-2—targeting all endogenous UBE2D enzymes and ‘siCTRL’ targeting an absent ectopic enzyme, luciferase [12].

**GFP-Trap bead-assisted immunoprecipitation.** U2OS-derived cells were seeded in three 150 mm dishes (Corning; Corning, NY, USA) per condition. The cells were then induced with doxycycline (wt—15 μg mL^−1^, CD—0.15 μg mL^−1^) overnight. After 16–24 h, the GFP expression was checked using fluorescence microscopy followed by immunoprecipitation (IP). The total GFP-Trap bead slurry (Chromotek; Munich, Germany; cat#gtma-100) was washed in IP buffer (10% glycerol, 20 mM Tris/HCl—pH 7.5, 40 mM NaCl, 2 mM MgCl_2_, 0.5% Nonidet P-40, 1x cOmplete protease, 1x phosphatase inhibitor, and 10 mM N-ethylmaleimide) three times. The beads were then added to low-binding microcentrifuge tubes (Corning; Corning, NY, USA; CoStar; 1.7 mL capacity; hereafter referred to as CLBs) on ice. The following steps were performed on ice. The replicate dishes were washed once in ice-cold PBS. Cells were then lysed using IP buffer (with added 1:100 benzonase; Sigma Aldrich; St. Louis, MO, USA) and collected. The replicate lysates were pooled. Once combined, the lysate was made up to 1 mL with IP buffer, to which aqueous sodium chloride solution (5 M, 100 μL) was added. This mixture was briefly agitated and then centrifuged (17,000× *g*, 0.5 h, 4 °C). Protein concentration of the samples was determined using a Bradford assay, and then normalised. A portion of the supernatant (5%) was reserved at this stage and kept on ice. The remaining supernatant was transferred to a CLB containing the beads. The CLBs, with supernatant and beads, were rotated slowly for 1 h (4 °C, 10–15 rpm). Then, a portion of the supernatant (5%) was reserved and kept on ice. The beads were washed twice with IP buffer and three times with high-salt IP buffer (as above but 500 mM NaCl). The beads were then centrifuged (2000× *g*, 2 min.) and the supernatant removed. SDS PAGE sample buffer was added and the mixture heated at 95 °C for 7 min before gel loading for SDS-PAGE.

**Clonogenic survival.** U2OS cells were seeded in the inner 8 wells of 24-well plates (500 cells per well). The following day, the plates were treated with requisite drug concentrations (diluted in growth medium). *N*-AC pre-treated plates were treated with *N*-AC-containing growth medium (10 mM, filter-sterilised) for 1 h prior to drug treatment. After 7 days, the plates were stained with crystal violet/ethanol (0.5% *w*/*v* crystal violet, 20% *v*/*v* ethanol) solution and imaged using a ChemiDoc (Bio-Rad; Hercules, CA, USA) instrument. The images were quantified using the ImageJ (RRID: SCR_003070; Bethesda, MD, USA) plug-in ‘ColonyArea’ [65].

**Live cell imaging.** Cells were seeded in 96-well plates (Greiner Bio-One; Frickenhausen, Germany) 24 h before drug treatment (2500 cells per well for GFP-H2B OVCAR3 cells; 3000 cells per mL for GFP-H2B Kuramochi and GFP-H2B COV318 cells). The cells were treated with the requisite concentration of drug diluted in growth medium and further incubated (2 h), after which point the designated plates were irradiated (2 Gγ) using a Faxitron CellRad irradiator (Faxitron Bioptics, LLC; Tucson, AZ, USA). Cells were imaged utilising an IncuCyte ZOOM or IncuCyte S3 (Essen BioScience; Ann Arbor, MI, USA; RRID: SCR_019874) system, at 20x magnification (Nikon; Tokyo, Japan), in a humidified, 5% CO_2_ atmosphere at 37 °C every 4 h. Real-time, automated analysis was completed to quantify green fluorescent object count (GOC) corresponding to a precise cell count of the GFP-H2B modified cell lines. Data were exported from IncuCyte software, processed in Microsoft Excel, and graphed using Prism 8 (GraphPad; San Diego, CA, USA; RRID: SCR_002798). IC_50_ calculations were performed using calculations and transformations in Prism 8 (GraphPad; San Diego, CA, USA; RRID: SCR_002798). The area under a normalised curve (AUC; baseline of *y* = 0) was calculated for each experimental condition. The values generated were normalised to a vehicle-treated control (DMSO). Subsequently, the normalised values were plotted against the log of drug concentrations in a non-linear regression curve analysis via the least squares (ordinary fit) method.

**High-content screening and immunofluorescence.** U2OS cells were seeded into 96-well plates (Perkin Elmer; Waltham, MA, USA; cat#6005550) 24 h before drug treatment (15,000–20,000 cells per well). The cells were treated with drug(s) diluted in growth medium and incubated for the indicated times (if not indicated: 2 h), at which point designated plates were irradiated (2 Gγ, recovery 0.5 h) as above. For pre-extracted samples (γH2AX, 53BP1, conjugated ubiquitin (FK2)): growth medium was removed, the cells pre-extracted by incubating them for 10 min at room temperature (RT) in 25 mM HEPES pH 7.4, 50 mM NaCl, 3 mM MgCl_2_, 0.5% Triton X-100 (ITW reagents; Chicago, IL, USA), 300 mM D-sucrose), and then fixed in 2% paraformaldehyde in phosphate-buffered saline (PBS; 20 min, RT) and subsequently, 0.1% Tween-20 (ProMega; Madison, WI, USA) in phosphate-buffered saline (hereafter referred to as PBST). PBST washes (3×) were performed before and after each step. For non-pre-extracted samples (RPA1 immunofluorescence): growth medium was removed, the cells fixed in 2% paraformaldehyde in PBS (20 min, RT), permeabilised using 0.5% Triton X-100 in PBS (15 min, RT), and then stored in PBST (250 uL per well) at 4 °C. PBST washes (3x) were performed before and after each step. For both pre-extracted and non-extracted samples, cells were then stained using primary antibodies (γH2AX, Merck Millipore; Burlington, MA, USA; cat#05-636, RRID: AB_309864—1:500, 53BP1, Novus; St. Louis, MO, USA; cat#NB100-034—1:1000, FK2, Enzo Lifesciences; Farmingdale, NY, USA; cat#BML-PW8810, RRID: AB_10541840—1:250, RPA1, Abcam; Cambridge, UK; cat#ab79398, RRID: AB_1603759—1:500; 1 h incubation at RT) and the corresponding rabbit or mouse-derived secondary antibody (anti-rabbit Alexa Fluor 488 IgG (H+L) (Invitrogen; Waltham, MA, USA; cat#A27034, RRID: AB_2536097), anti-mouse Alexa Fluor 488, and anti-mouse Alexa Fluor 594 IgG (H+L) (Invitrogen; Waltham, MA, USA; cat#A28175, RRID: AB_2536161, and cat#A11032, RRID: AB_2534091)—1:500 as supplied, with DAPI—1:1000 from aforementioned DMSO stock, 1 h incubation at RT, obscured from light) in blocking buffer (5% bovine serum albumin; Sigma Aldrich; St. Louis, MO, USA; in PBST). After each antibody incubation, cells were washed in PBST (3x). Images were acquired using Operetta or Phenix high-content imaging systems (Perkin Elmer; Waltham, MA, USA) and quantified using Columbus high-content imaging and analysis software (Perkin Elmer; Waltham, MA, USA). Nuclear mean fluorescence intensity or nuclear foci number were quantified as values per cell, using Columbus building blocks. A mean value per imaged field was then calculated and graphed using Prism 8 (GraphPad; San Diego, CA, USA). GFP-UBE2D1 wt/CD U2OS-derived cell lines were grown in glass-bottom dishes (35 mm; IBIDI; Fitchburg, WI, USA), before inducing with doxycycline as described in the immunoprecipitation procedure above. The cells were then processed in accordance with the non-pre-extracted immunofluorescence protocol specified above. Once fixed and permeabilised, the cells were stained using anti-GFP primary antibody (1:500) followed by anti-mouse Alexa Fluor 488 secondary antibody as above. Images were acquired using the Invitrogen EVOS FL Auto 2 Imaging System (Thermo Scientific; Waltham, MA, USA) and associated software.

**Antibodies**. The following antibodies were used for immunofluorescence: anti-γH2AX (Merck Millipore; Burlington, MA, USA; cat#05-636, RRID: AB_309864), anti-53BP1 (Novus; St. Louis, MO, USA; cat#NB100-034), anti-FK2 (Enzo Lifesciences; Farmingdale, NY, USA; cat#BML-PW8810, RRID: AB_10541840), anti-GFP (Roche; Basel, Switzerland; cat#11814460001, RRID: AB_390913), anti-RPA1 (Abcam; Cambridge, UK; cat#ab79398, RRID: AB_1603759), anti-rabbit Alexa Fluor 488 IgG (H+L) (Invitrogen; Waltham, MA, USA; cat#A27034, RRID: AB_2536097), anti-mouse Alexa Fluor 488, and anti-mouse Alexa Fluor 594 IgG (H+L) (Invitrogen; Waltham, MA, USA; cat#A28175, RRID: AB_2536161, and cat#A11032, RRID: AB_2534091). They were diluted and used as described above. The following primary antibodies were used for immunoblotting (10–16 h incubation at 4 °C): anti-FK2 (Enzo Lifesciences; Farmingdale, NY, USA; cat#BML-PW8810, RRID: AB_10541840—1:500 in 1 % BSA in TBST 1×), anti-GFP (Roche; Basel, Switzerland; cat#11814460001, RRID: AB_390913—1:1000 in 5% BSA in TBST 1×), anti-γH2AX (Merck Millipore; Burlington, MA, USA; cat#05-636, RRID: AB_309864—1:2000 in 1% BSA in TBST 1×), anti-H2AX (Abcam; Cambridge, UK; cat#ab11175, RRID: AB_297814—1:15,000 in 1% BSA in TBST 1×), anti-RPA1 (Abcam; Cambridge, UK; cat#ab79398, RRID: AB_1603759—1:1000 in 5% BSA in TBST 1×), anti-UBE2D1 (Abcam; Cambridge, UK; cat#ab176561—1:10,000 in 1% BSA in TBST 1×), and anti-UBE2D3 (Cell Signalling Technology; Danvers, MA, USA; cat#4330, RRID: AB_10544697—1:1000 in 1% BSA in TBST 1×). The following secondary antibodies were used for immunoblotting (1 h, RT): anti-rabbit or anti-mouse horseradish-peroxidase-conjugated secondary antibody (Dako Agilent; Santa Clara, CA, USA; —1:10,000 in 1% BSA in TBST 1×, 1 h incubation at RT).

**SDS-PAGE and immunoblotting.** SDS-PAGE was performed using 15% Tris/glycine gels that were prepared in-house or NuPAGE 4–12% Bis-Tris pre-cast gels (Invitrogen; Waltham, MA, USA; cat#NP0321BOX) following the manufacturer’s guidelines. For immunoblotting, the resolved gels were electroblotted onto PVDF membranes (GE healthcare; Chicago, IL, USA), blocked (5% BSA in TBST-50 mM Tris, pH 7.6, 150 mM NaCl, 0.1% Tween-20, RT, 0.5–1 h), and then stained with the appropriate antibodies as indicated above. The membranes were visualised on a ChemiDoc system (Bio-Rad; Hercules, CA, USA), using Amersham (Amersham, UK) enhanced chemiluminescence detection reagents according to manufacturer’s instructions.

**Flow cytometry**. For DNA content analysis, cells were treated as indicated and then harvested according to experimental condition. Cells were washed in PBS, then counted. All samples were resuspended in a small volume of PBS and gently vortexed while slowly adding 70% ethanol. Cells were fixed overnight (4 °C). Following this, samples were washed in PBS and then resuspended in 0.5% Triton X-100 in PBS, RNase A (200 μg mL^−1^; Sigma Aldrich; St. Louis, MO, USA), and propidium iodide (20 μg mL^−1^; Sigma Aldrich; St. Louis, MO, USA), before incubation (2 h, 4 °C). Finally, cells were resuspended in PBS and stored (4 °C) prior to analysis or analysed immediately using an Invitrogen Attune NxT flow cytometer (Thermo Fisher Scientific; Waltham, MA, USA). A total of 10,000 cells were analysed per condition using FlowJo (LLC; Ashland, Oregon, United States; RRID: SCR_008520).

**Statistical analysis.** All statistical analysis was performed using Prism 8 (GraphPad; San Diego, CA, USA; RRID: SCR_002798) and Excel (Microsoft; Redmond, WA, USA; RRID: SCR_016137). Unless otherwise stated, data were used as generated or a mean (±SEM) was calculated based on technical and/or biological replicate experiments. Details of statistical analyses are described in the Figure legends. Unless otherwise stated, statistical significance between two groups was determined by two-tailed Student’s t-test, and between three or more groups using one-way ANOVA and Tukey’s multiple comparisons test for which the multiplicity adjusted p-value is reported as n.s.—*p* > 0.05, *—*p* < 0.05, **—*p* < 0.01, ***—*p* < 0.001, and ****—*p* < 0.0001. Further statistical analyses, such as non-regression curves and IC_50_ determination were performed using Prism 8′s (GraphPad; San Diego, CA, USA; RRID: SCR_002798) internal transformations.

**Replication stress signatures.** Gene expression heat map was created using RNAseq TPM gene expression values according to cell line as given in DepMap Portal (RRID: SCR_017655; Public 21Q2 release, “CCLE_expression.csv”) [66]. Values are Log_2_ transformed, using a pseudo-count of 1. Published values were graphed using Prism 8 (GraphPad; San Diego, CA, USA; RRID: SCR_002798). RAD51D was included in addition to the genes specified in the cited work [34].

## 5. Conclusions

The sesquiterpene lactones, BdS and ATL, potentiate the effects of olaparib-mediated PARP-trapping, resulting in increased RPA consumption, and DNA damage in HR-proficient, p53 wildtype U2OS cancer cells. Over longer treatment durations, combination treatments induce synergistic rises in pan-γH2AX nuclear staining, 53BP1 foci, and mitotic defects, consistent with replication stress and catastrophe. Along with the established ROS inducer, ATL [14], the effects of BdS were attenuated by the antioxidant, N-acetyl cysteine, while covalent binding capacity also proved essential for compound activity, as shown by the analogue, BdS-H_2_. Cell survival assays recapitulated these effects with low doses of the individual compounds, and survival was further reduced with the addition of a WEE1 inhibitor. In conjunction with cell-cycle analysis of the treated cells, this suggested that the G2 cell-cycle stalling, likely p53-mediated, that occurs in the absence of WEE1 inhibition is protective to cell fate. These novel findings indicate that the targeted covalent inhibition of cellular redox regulators, such as thioredoxin reductases, warrants further research and could be employed to extend the use of PARP-trapping small molecules to HR-proficient, p53 wildtype cancers that exhibit a high basal level of ROS and/or an intrinsic susceptibility to replication stress.

## Data Availability

The data presented in this study are available upon request from the corresponding authors.

## Supplementary Materials

The following supporting information can be downloaded at: https://www.mdpi.com/article/10.3390/ijms23031116/s1.
