# Supplementary material for: Sesquiterpene Lactones Potentiate Olaparib-Induced DNA Damage in p53 Wildtype Cancer Cells
Source: Int J Mol Sci. 2022 Jan 20;23(3):1116. doi: 10.3390/ijms23031116 (PMC8835362; doi:10.3390/ijms23031116)
Supplement: Supplementary file 1 [file ijms-23-01116-s001.zip › 2022-01-18_IJMSsupps.pdf]

# Supplementary Figure S1

A

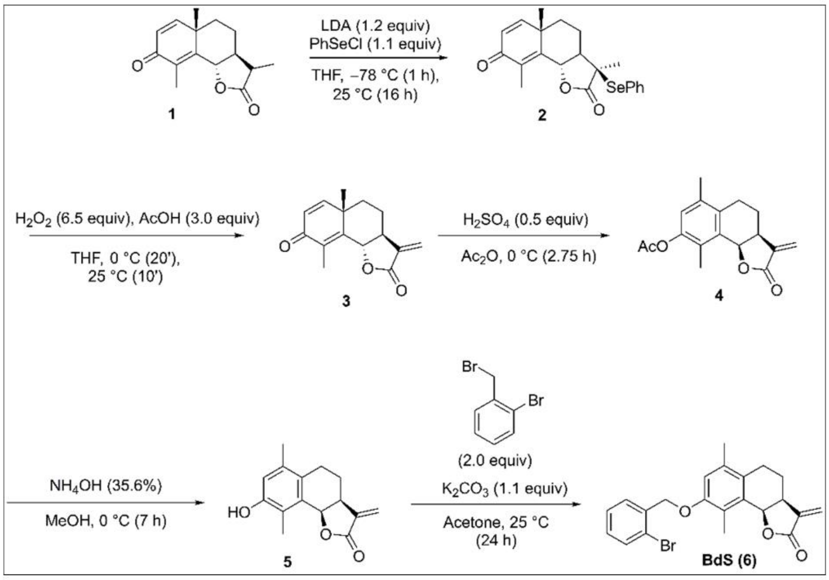


B

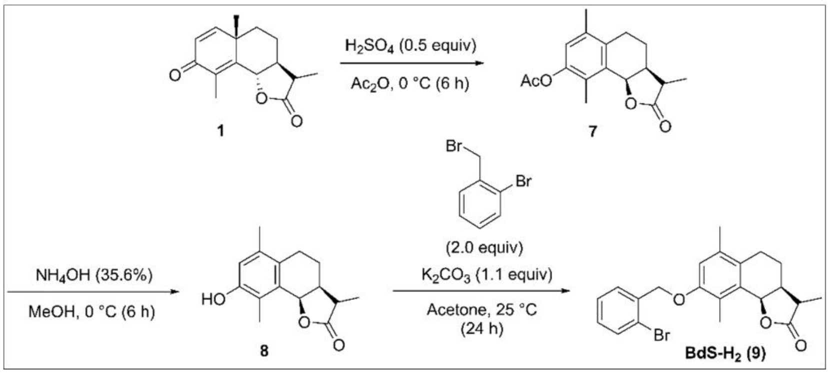


C

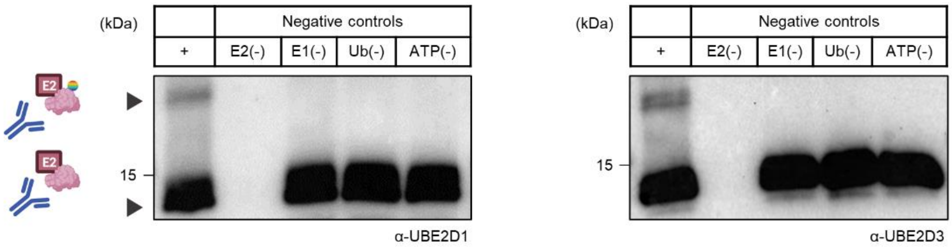


D

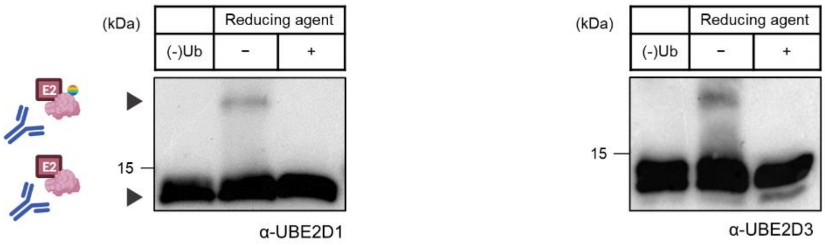

**Supplementary Figure S1. Synthesis of BdS and BdS-$H_2$, and *in vitro* ubiquitylation assay workflow controls.** (**A**) Synthetic route towards sesquiterpene lactone, BdS, as previously described [21]. (**B**) Synthetic route towards negative control compound, BdS-$H_2$. (**C**) Systematic negative controls for the *in vitro* ubiquitylation assays performed for UBE2D1 (left, $n$ = 1), and UBE2D3 (right, $n$ = 1), with all the components being required for ubiquitin-loading of the relevant E2 as indicated by the formation of the relevant band in the left-most lane (+). (**D**) Representative blots of a control experiment demonstrating that ubiquitin-loaded E2 formation is abrogated with the addition of reducing agent (β-mercaptoethanol) for both UBE2D1 ($n$ = 2 independent experiments) and UBE2D3 ($n$ = 2 independent experiments).

## Supplementary Figure S2

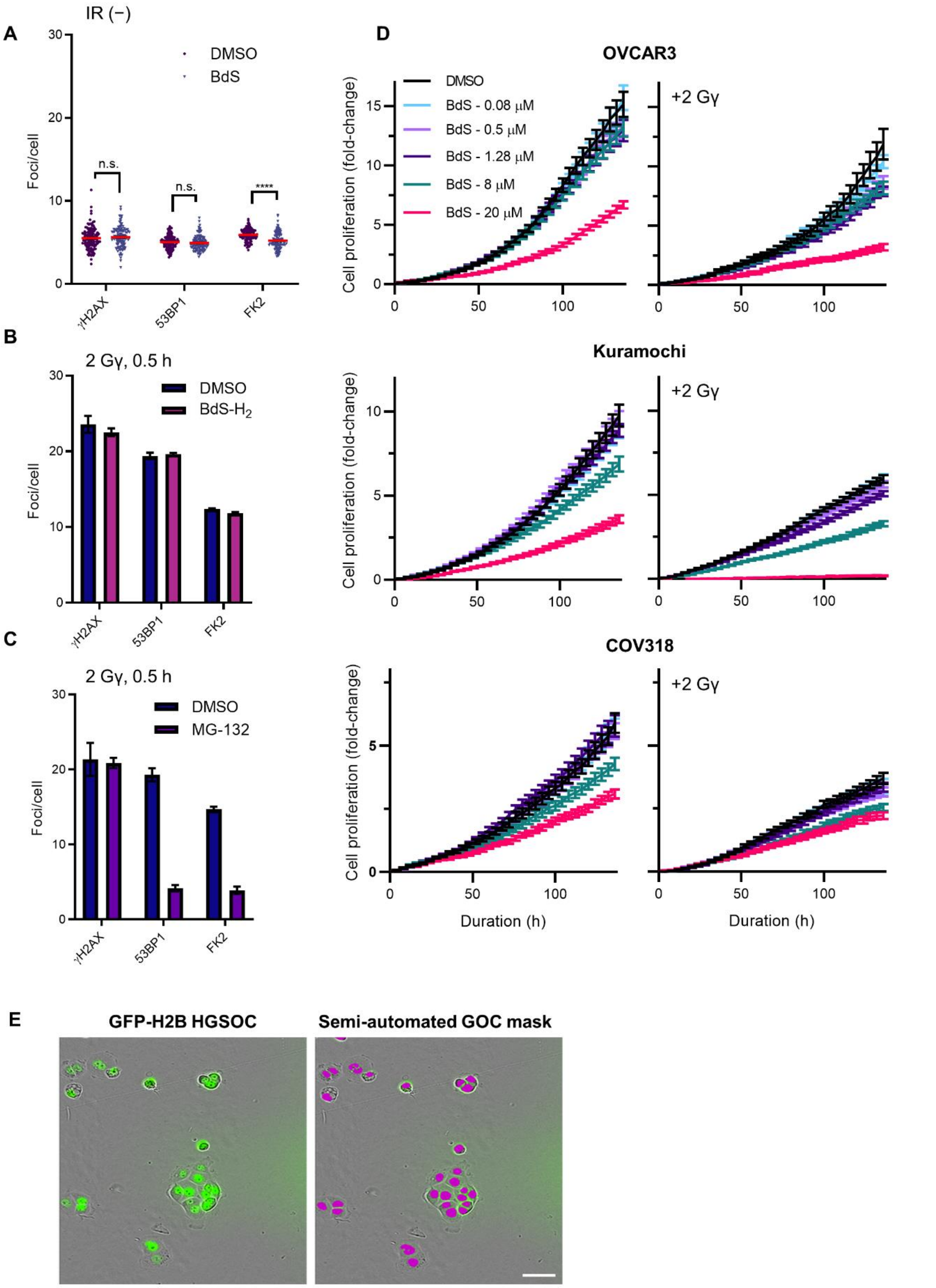

**Supplementary Figure S2**. **Establishing DNA repair and HGSOC cell growth workflows.** (**A**) Quantification of foci number per cell for DNA damage response (DDR) factors (γH2AX, 53BP1, FK2), omitting irradiation for U2OS cells treated with BdS (20 μM, 2 h). Each dataset represents a minimum of 3,000 cells (3 replicates; *n* = 126; mean ± SEM). Data points correspond to each recorded field. (**B**) Quantification of foci number per cell for DDR factors (γH2AX, 53BP1, FK2), following irradiation (2 Gγ, 0.5 h recovery) and treatment with BdS-$H_2$ (200 μM, 2 h). Each dataset represents a minimum of 1,500 U2OS cells (2 replicates; *n* = 2; mean ± SEM). (**C**) Quantification of foci number per cell for DDR factors (γH2AX, 53BP1, FK2), following irradiation (2 Gγ, 0.5 h recovery) and treatment with proteasome inhibitor, MG132 (10 μM, 2 h). Each dataset represents a minimum of 1,500 U2OS cells (2 replicates; *n* = 2; mean ± SEM). (**D**) Representative fold-change growth curves (*n* = 27 for each data point) for high-grade serous ovarian carcinoma (HGSOC) cell lines in the presence of BdS over 136 h – top to bottom: OVCAR3, Kuramochi, COV318. Left to right: non-irradiated, irradiated (2 Gγ) for each cell line. (**E**) Representative images of the green object count (GOC) mask applied to GFP-H2B HGSOC live cell imaging in order to track proliferation. Scale – 50 μM. Statistical significance indicated as follows: n.s.—p > 0.05, and ****—p < 0.0001.

## Supplementary Figure S3

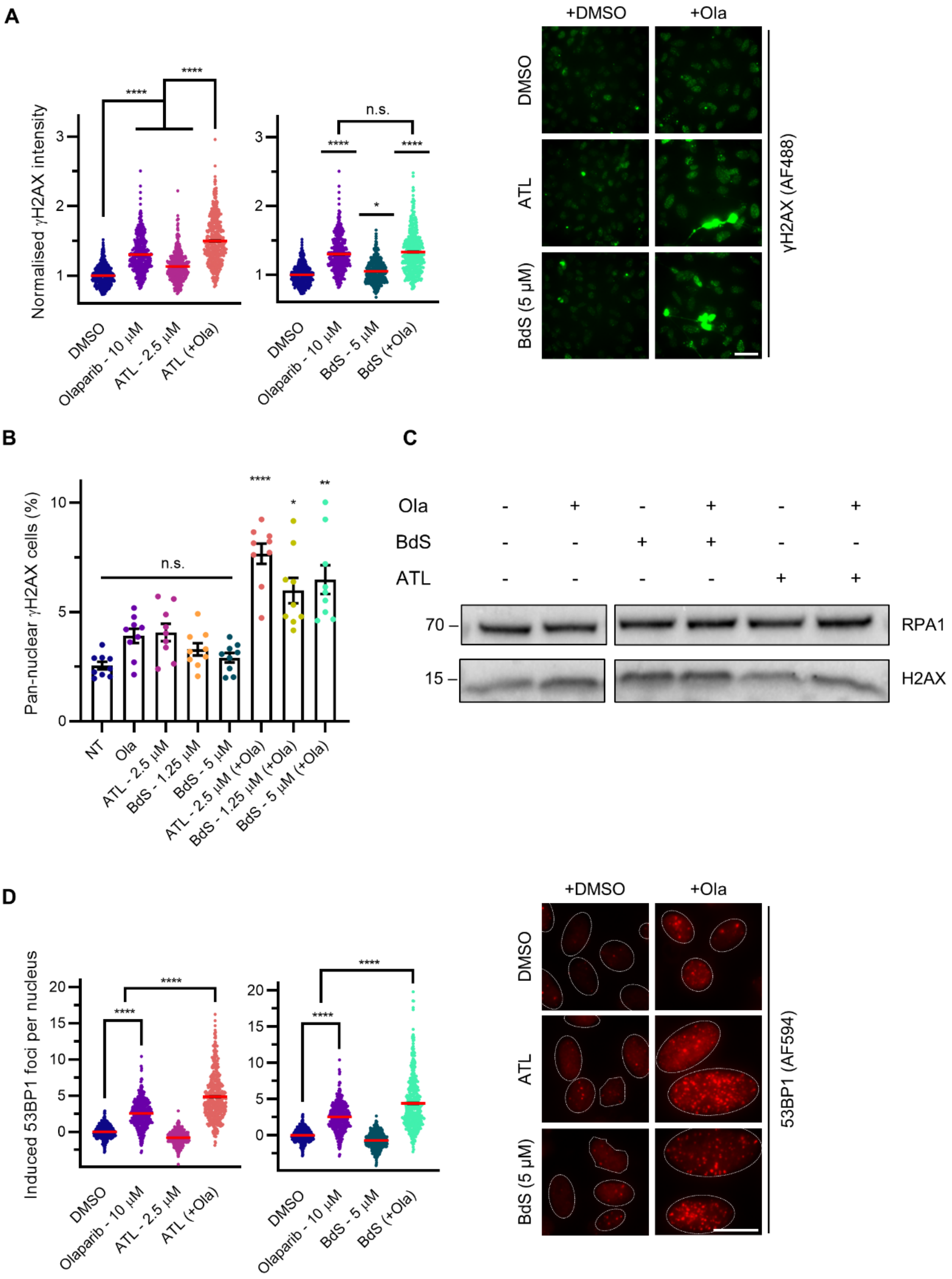

**Supplementary Figure S3**. **DNA damage markers characterise cellular response to combination treatments over longer durations.** (**A**) Quantification (left) and representative images (right, Alexa Fluor 488; AF488) of normalised nuclear γH2AX immunofluorescence intensity (to vehicle-only – DMSO-treated cells) after 72 h treatment with the specified compounds alone or in combination. Each dataset represents a minimum of 15,000 U2OS cells (3 independent experiments; *n* = 504; mean ± SEM), while data points correspond to each recorded field. Scale bar – 50 μm. (**B**) Quantification of pan-nuclear γH2AX immunofluorescence at 72 h. Each dataset represents a minimum of 4,500 U2OS cells (3 independent experiments; *n* = 9; mean ± SEM). (**C**) Immunoblot showing whole cell extract levels of RPA1, and H2AX following treatment of U2OS cells with olaparib (10 μM) alone, and in combination with alantolactone (ATL; 10 μM), and BdS (10 μM) for 72 h (*n* = 1). Images taken from the same membrane for RPA1 and H2AX, respectively. (**D**) Quantification (left) and representative images (right Alexa Fluor 594; AF594) of induced 53BP1 foci (compared to vehicle-only – DMSO-treated cells) after 72 h treatment with the specified compounds alone or in combination as indicated. Each dataset represents a minimum of 15,000 U2OS cells (3 independent experiments; *n* = 504; mean ± SEM), while data points correspond to each recorded field. Nuclei are outlined in white using DAPI as a reference. Scale bar – 20 μm. Statistical significance indicated as follows: n.s.—p > 0.05, *—p < 0.05, **—p < 0.01, and ****—p < 0.0001

## Supplementary Figure S4

**A**

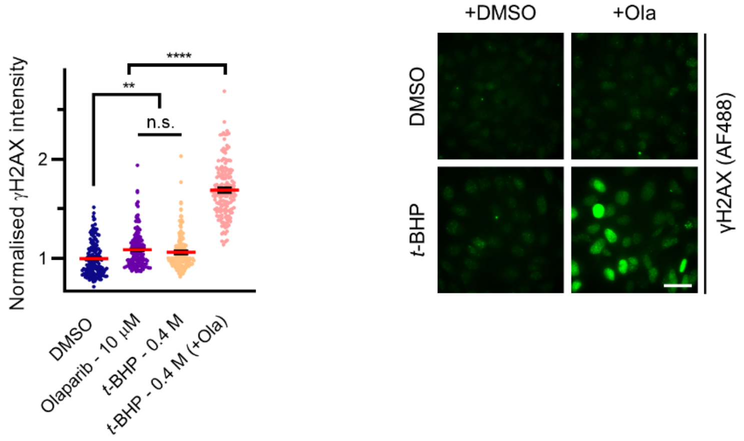


**B**

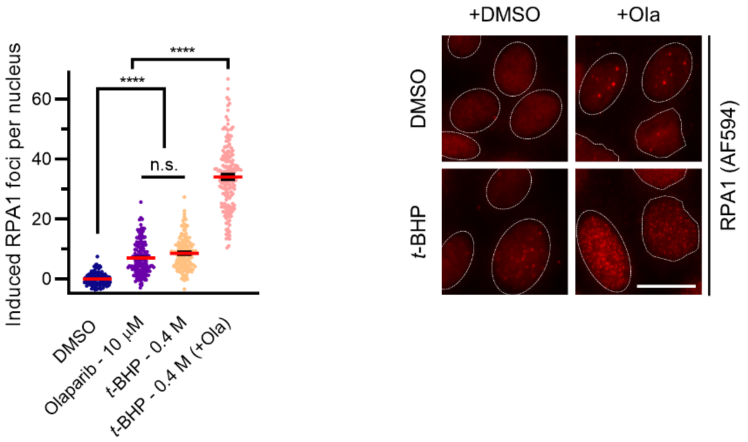


**Supplementary Figure S4**. **A combination of ROS inducer, *tert*-butyl hydroperoxide (*t*-BHP), and olaparib treatments phenocopies the effects seen with olaparib and ATL/BdS combination treatments.** (**A**) Quantification (left) and representative images (right, Alexa Fluor 488; AF488) of normalised nuclear γH2AX immunofluorescence intensity (to vehicle-only – DMSO-treated cells) after 24 h treatment with olaparib, *t*-BHP, or in combination as specified. Each dataset represents a minimum of 7,500 U2OS cells (3 replicates; *n* = 168; mean ± SEM), while data points correspond to each recorded field. Scale bar – 50 µm. (**B**) Quantification (left) and representative images (right) of induced RPA1 foci per cell (Alexa Fluor 594; AF594), after 24 h treatment with olaparib, *t*-BHP, or in combination as specified compared to vehicle only (DMSO). Each dataset represents a minimum of 7,500 U2OS cells (3 replicates; *n* = 168; mean ± SEM). Data points correspond to each recorded field (mean ± SEM). Nuclei are outlined in white using DAPI as a reference. Scale bar – 20 µm. Statistical significance indicated as follows: n.s.—$p > 0.05$, **—$p < 0.01$, and ****—$p < 0.0001$.
